# Selection on the Fly: Short-Term Adaptation to an Altered Sexual Selection Regime in *Drosophila pseudoobscura*

**DOI:** 10.1093/gbe/evad113

**Published:** 2023-06-21

**Authors:** Carolina Barata, Rhonda R Snook, Michael G Ritchie, Carolin Kosiol

**Affiliations:** Centre for Biological Diversity, University of St Andrews, St Andrews, UK; Institute of Science and Technology Austria, Klosterneuburg, Austria; Department of Zoology, Stockholm University, Stockholm, Sweden; Centre for Biological Diversity, University of St Andrews, St Andrews, UK; Centre for Biological Diversity, University of St Andrews, St Andrews, UK

**Keywords:** genomics, sexual selection, experimental evolution, time series, pooled sequencing

## Abstract

Experimental evolution studies are powerful approaches to examine the evolutionary history of lab populations. Such studies have shed light on how selection changes phenotypes and genotypes. Most of these studies have not examined the time course of adaptation under sexual selection manipulation, by resequencing the populations’ genomes at multiple time points. Here, we analyze allele frequency trajectories in *Drosophila pseudoobscura* where we altered their sexual selection regime for 200 generations and sequenced pooled populations at 5 time points. The intensity of sexual selection was either relaxed in monogamous populations (M) or elevated in polyandrous lines (E). We present a comprehensive study of how selection alters population genetics parameters at the chromosome and gene level. We investigate differences in the effective population size—$N_{e}$—between the treatments, and perform a genome-wide scan to identify signatures of selection from the time-series data. We found genomic signatures of adaptation to both regimes in *D. pseudoobscura*. There are more significant variants in E lines as expected from stronger sexual selection. However, we found that the response on the X chromosome was substantial in both treatments, more pronounced in E and restricted to the more recently sex-linked chromosome arm XR in M. In the first generations of experimental evolution, we estimate $N_{e}$ to be lower on the X in E lines, which might indicate a swift adaptive response at the onset of selection. Additionally, the third chromosome was affected by elevated polyandry whereby its distal end harbors a region showing a strong signal of adaptive evolution especially in E lines.

**Significance**

Experimental evolution has served as a tool to describe signatures of sexual selection in the genome. Here, we analyzed allele frequencies changes in *Drosophila pseudoobscura* populations whose mating system was altered during the course of an experimental evolution study. We found that monogamy and polyandry regimes both show signs of adaptation suggesting that the strength of directional sexual selection was sufficient to overcome genetic drift in our small populations. Our results have helped to improve our understanding of adaptation to different sexual selection intensities.

## Introduction

Evolutionary biologists have put considerable effort into uncovering how social environments shape evolution, especially those that change sexual selection pressures. Studies over the years have found differences in courtship phenotypes as well as other fitness-related traits caused by altered mating systems (Chapman et al. 1995; Wigby and Chapman 2004; Chenoweth and Blows 2005; Hollis et al. 2017). Due to the effects of mate competition, male harm has also been found to evolve under specific environmental conditions (Holland and Rice 1999; Yun et al. 2019).

A few key studies have tried to identify the genetic basis of adaptation to a new sexual selection regime and it has been suggested that this may often involve sexually antagonistic variation. Sexually antagonistic loci were initially hypothesized to be more prevalent on the X chromosome (Rice 1984). In a model with equal dominance in both sexes, Rice proposed that a sexually antagonistic variant that is either dominant and female-beneficial or recessive and advantageous to males should increase in frequency. This would then result in X-linked sexually antagonistic variation invading more readily when compared to autosomal loci. Rice’s prediction was confirmed in several studies (e.g., Chippindale et al. 2001; Innocenti and Morrow 2010). However, subsequent theoretical predictions suggested that autosomes are just as likely to harbor sexually antagonistic polymorphism as the X under certain conditions, especially when relaxing the assumption of parallel dominance between the sexes (Fry 2009; Ruzicka and Connallon 2020). Other studies have subsequently shown that sexual selection seems to affect many of the same genomic regions as those affected by natural selection regardless of chromosomal location (Chenoweth et al. 2015). Perhaps surprisingly, the X chromosome was found not to be a hotspot for sexually antagonistic variation in lines of *Drosophila melanogaster* (Ruzicka et al. 2019). We also know that sexual conflict can be resolved by changes in gene expression in response to sexual selection (Innocenti and Morrow 2010; Hollis et al. 2014). Evidence indicates that sexual antagonism can lead to sex-biased gene expression within a relatively short timescale (Wright et al. 2018). The importance of sexual selection in shaping the genomic landscape of a population is therefore still largely undiscovered. Here, we characterize the adaptive response of polymorphic sites throughout the genome in response to experimental variation in sexual selection. Thus, we can address emerging patterns in response to short-term adaptation to either relaxation of or the presence of sexual selection.

We investigate patterns of genetic adaptation of *Drosophila pseudoobscura* flies in a socially manipulated environment across 200 generations of evolution. The experiment consisted of rearing replicated populations under either monogamy—M—or elevated polyandry—E. These two treatments should relax or increase sexual selection, respectively. It has been shown that behavioral and physiological traits have diverged between these lines throughout the experiment. These include courtship song and male mating and courtship rates. In summary, E males produced more attractive song, show decreased singing latency and faster songs over longer periods of time (Snook et al. 2005; Debelle et al. 2017). These males also had higher courtship rates (Crudgington et al. 2010). In contrast, M males had smaller accessory glands and sired fewer progeny (Crudgington et al. 2009). Interestingly, female preference also seems to have coevolved with male signals in opposite directions between the two selection regimes (Debelle et al. 2014).

These earlier studies have demonstrated that sexual selection substantially affected multiple traits as populations adapted. However, a better understanding of the genetic mechanisms responsible for differences in phenotype is needed. Analyses of gene expression patterns in virgin M and E females showed that 14% of the transcriptome was differentially expressed (Immonen et al. 2014) and 70% of these differences were sex biased. This suggests that loci under sexually antagonistic selection might be contributing to divergence between the treatments. The majority of differentially expressed genes was found in males’ heads, which is consistent with the importance of behavioral traits (Veltsos et al. 2017). Conversely, M treatment flies were predicted to exhibit a feminization of the transcriptome. In M populations, there was indeed a feminization of male heads but, contrary to expectations (Haig 2006; Hollis et al. 2014), male abdomens and both female heads and abdomens were masculinized. This is important since the abdomens house the sex-specific reproductive tissues. Gene expression differences are thus well characterized in this system, but there is a clear gap in regard to the population genomics associated with altering *D. pseudoobscura*’s mating system.

There are theoretical predictions on the genetic basis of adaptation to an altered mating system that we can consider. First, diversity on the autosomes (*A*) is expected to differ from *X*-linked diversity due to differences in the effective population size. Under M, *X*/*A* diversity ratios are predicted to be roughly 3/4. Because males only carry one copy of the X chromosome, $N_{eX}=\frac{3}{4}N_{eA}$ under the assumption of equal variance in reproductive success between the sexes. This affects the efficacy of selection and, consequently, diversity ratios. Under polyandry, however, these ratios are expected to shift towards even lower values, especially if populations are founded following a bottleneck (Pool and Nielsen 2008). Our experimental design tried to counter-act this effect: the family size for E and M populations was set to ensure that $N_{e}$ on the autosomes was roughly the same in both lines (addressed in Snook et al. 2009). In addition, if most beneficial mutations on the X chromosome are partially recessive, diversity on the X is predicted to be lower compared to that on the autosomes (Betancourt et al. 2004; Vicoso and Charlesworth 2009). These effects combined with sexual selection pressures are expected to result in a marked reduction in diversity on the X chromosome. Transcriptome evolution seems to be a large part of the adaptive response to sexual selection (Connallon and Knowles 2005; Hollis et al. 2014). In *Drosophila melanogaster*, the X chromosome is known to be enriched in female-biased genes (Meiklejohn and Presgraves 2012) and significantly depleted in male-biased genes (Parisi et al. 2003), which might influence mating success. However, some important fertilization success genes that are male biased are located on the X (Hughes and Leips 2006; Greenspan and Clark 2011). From evidence in *D. melanogaster*, one would predict sexual selection signatures in *D. pseudoobscura* to be especially prominent on the X where genes that are key for successful mating are located.

In addition to the genomic location of the variants that may respond to sexual selection, it is still unclear how it can cause allele frequencies to change in the short term. More importantly, much uncertainty still exists about the shape of those allele frequency trajectories during experimental evolution. Genomic time-series data can provide a missing link between phenotypic changes and proof of selection acting on the genome. For this reason, investigating allele frequency trajectories alongside experimental phenotypes in an Evolve & Resequence (E&R) design can prove very useful. They can help determine the rate and strength of selection acting on standing genetic variation that is driving genomic responses. Experimental populations are typically sampled and resequenced repeatedly within a certain number of generations. Samples at two time points can be used to test for selection by finding allele frequency changes (AFCs) that differ significantly between treatments (e.g., Pearson’s chi-square, $\chi^{2}$, test as in Griffin et al. 2017; Fisher’s exact test as in Burke et al. 2010; or the Cochran–Mantel–Haenszel, CMH, test as in Barghi et al. 2017). However, such approaches lack the ability to take advantage of the allele’s frequency trajectory. In contrast, more probabilistic modeling frameworks use time-series data to fully describe frequency trajectories. In particular, time-series approaches (e.g., Bait-ER—Barata et al. 2023) gain a lot from accounting for sampling noise typical of E&R experimental designs.

With a more sophisticated statistical framework, we can now characterize temporal allele frequency changes caused by sexual selection in these *D. pseudoobscura* populations. Here, we looked for evidence of adaptation in M and E line females both at the chromosome and gene level. Previously, Wiberg et al. (2021) examined genomic variation between the two treatments after ≈160 generations of selection and found “islands” of differentiation between the lines located on the X and third chromosomes. Our work here offers a more comprehensive analysis of full allele frequency trajectories. While we could not resequence before generation 21 due to lack of samples, we produced and analyzed a pooled sequencing (pool-seq) time series consisting of 5 time points throughout those 15 years of evolution in the lab. Starting at generation 21, this time series allows us to better understand both short-term and long-term effects. Our study assumes that adaptation has proceeded from standing genetic variation in these populations so that the effect of new mutations is negligible. We estimated the effective population size—$N_{e}$—which will be influenced by census size, mating system, and strength of selection for the four main *D. pseudoobscura* chromosomes: 2, 3, 4 and X. We used a Bayesian modeling approach—Bait-ER (Barata et al. 2023)—to infer selection on individual single nucleotide polymorphisms (SNPs) that allows for finding potential targets of selection. We combined individual SNP tests for selection with window-based estimates of the effective population size which gave us a clearer view of the rate of adaptation throughout the experiment. We examined $\frac{N_{eX}}{N_{eA}}$ ratios throughout the experiment to help illustrate how adaptation differed between the treatments. Finally, we explored the genomic location of the strongest signatures of selection at the chromosome and gene level.

## Results

### Diversity and AFCs

**Fig. 1.**
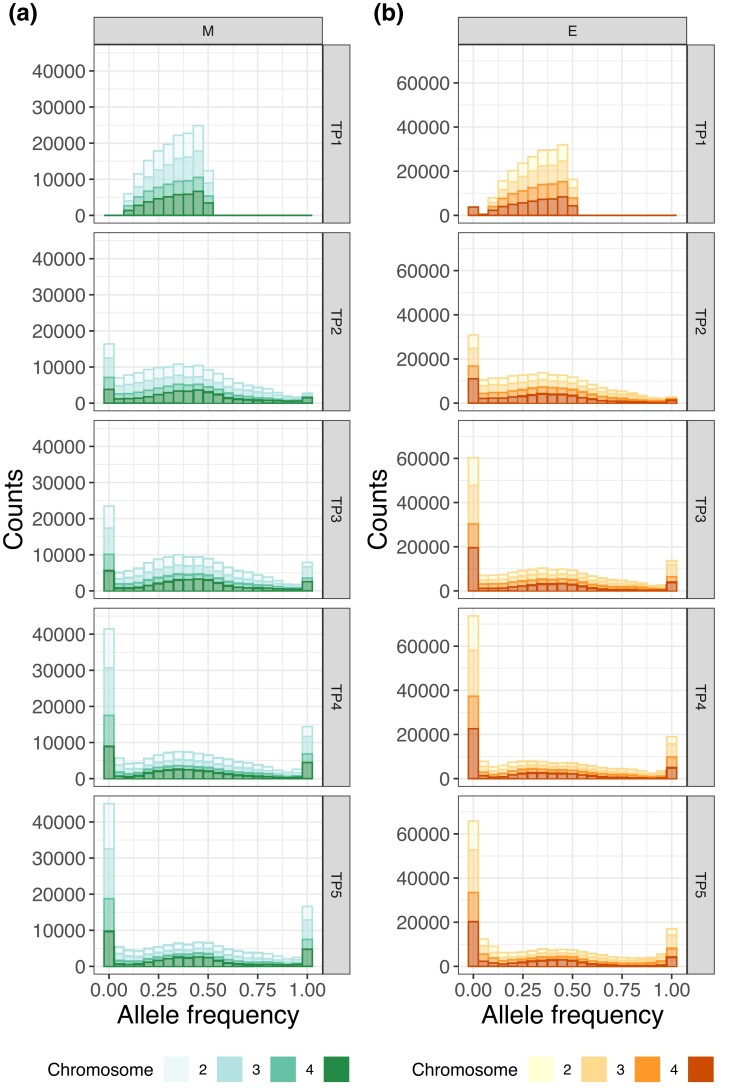
Allele frequency spectra in M and E populations. Allele frequency spectra in (*a*) for M and (*b*) for E populations per time point (rows) for each replicate population (columns). Each chromosome is colored in a different shade of green (M) or orange (E) as seen on the bottom legend.

We first investigated the allele trajectories by looking at allele frequency spectra throughout the experiment. The time series consists of 5 time points from generation 21 to generation 200 (T1: 21–22; T2: 59–63; T3: 112–116; T4: 160–164; T5: 200; see supplementary table S1, Supplementary Material online for more details). Frequency spectra at the start are flat distributions with maxima roughly at 0.45–0.55 (fig. 1). Alleles fixed at high rates, with the most fixations between time point 3 and time point 4 for M lines and time points 2 and 3 for E lines. Up to 29.5% and 16.9% more fixed sites than in the previous time point were observed for E and M, respectively. This indicates that diversity was more swiftly reduced in E populations, as expected if sexual selection is stronger in this regime. Allele frequency changes between first and last time point show distributions that are highly skewed towards low values (supplementary fig. S7, Supplementary Material online). This is especially true in the case of the X chromosome.

**Fig. 2.**
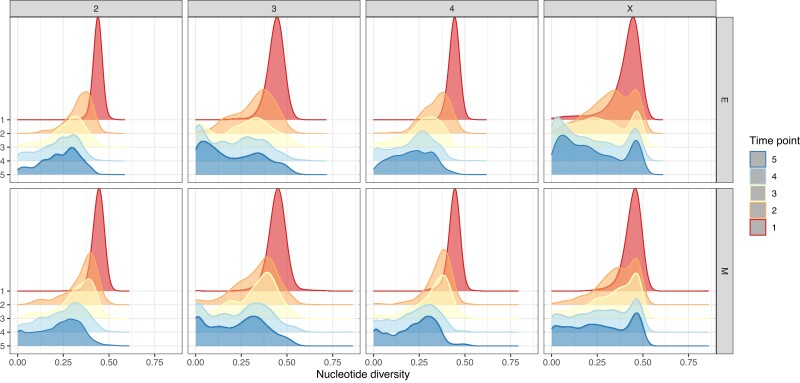
Relative π densities in M and E lines. Estimates were computed with Grenedalf (Czech and Exposito-Alonso 2021). Rows correspond to the two different treatments and columns to chromosomes. Each individual plot has five densities colored in as per side legend that correspond to one of the five time points.

Nucleotide diversity was measured in 250 kbp windows for each chromosome separately and at each time point. Diversity distributions show a marked reduction as time passes, particularly from time point 1 to time point 2 (fig. 2). All chromosomes’ densities peak at 0.4–0.5 per site at the first time point. At the end, densities for chromosomes 3 and X flatten out, especially for E flies. Interestingly, in M lines, π on the third chromosome becomes skewed towards very low values in later generations. In contrast, chromosomes 2 and 4 maintain more diversity. These results indicate that selection may have acted on the third and X chromosomes resulting in more windows of very low π across treatments. In other words, since genetic drift is expected, on average, to cause diversity to decrease evenly throughout the genome, low π found on the third and X chromosomes could be caused by the combined effect of selection and drift. Window estimates along each chromosome exhibit some diversity peaks, particularly on the third chromosome, that become flat towards the end of the experiment (supplementary fig. S8, Supplementary Material online).

**Fig. 3.**
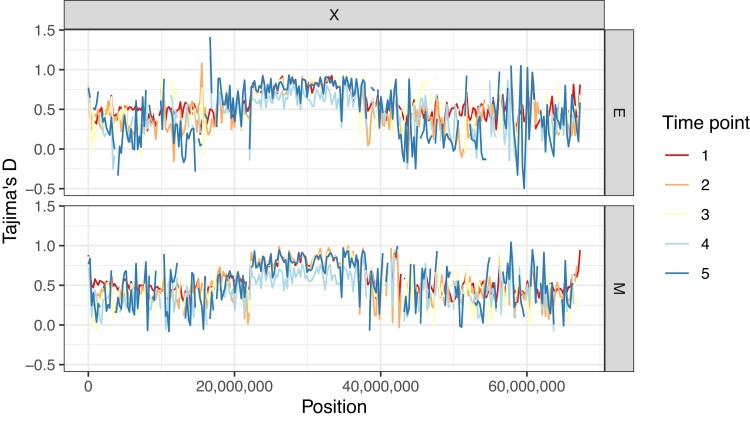
Tajima’s D estimates along the X chromosome for E and M lines. Rows correspond to the two different treatments. Estimates were calculated in 250k SNP windows with Grenedalf (Czech and Exposito-Alonso 2021). Lines are colored per time point according to the side legend.

Tajima’s D estimates throughout the genome are typically greater than 0 but show substantial variation amongst windows (fig. 3, supplementary fig. S9, Supplementary Material online). These were also computed for 250k SNP windows as for π. This result suggests that there is a lack of rare alleles in our data set which is unsurprising in a pool-seq experiment with stringent filtering criteria. As we filtered out variants with a minimum allele frequency of 0.025 at the first time point, very low frequency variants were removed. This can partly explain an elevated Tajima’s D at the start of the experiment. One noteworthy region, however, is the center of the X chromosome where Tajima’s D is consistently elevated in comparison to surrounding stretches (fig. 3). The pattern is present in both E and M populations throughout the whole experiment.

### Estimating the Effective Population Size

**Fig. 4.**
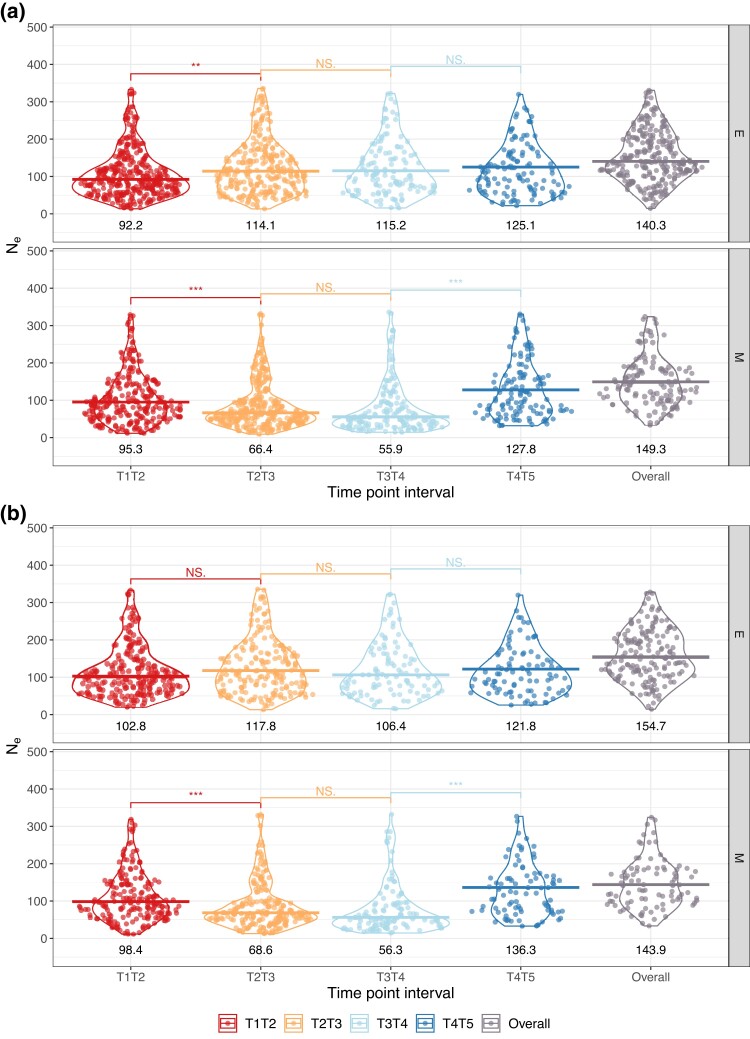
(*a*) Genome- and (*b*) autosome-wide $N_{e}$ estimates for M and E lines at different time-point intervals. Violin plots and data points are included for each time-point interval and treatment. Outliers were removed. Medians are shown as bars and median estimates can be found at the bottom of each violin plot. These were calculated using all 2k SNP window estimates from the four experimental replicates. “Overall” corresponds to $N_{e}$ estimates based on allele frequency changes between the first and last time point. Adjacent time-point intervals were compared using a Mann–Whitney *U* test. Significance level is indicated with *** for Pvalue<0.001, ** for Pvalue<0.01, * for Pvalue<0.05, and NS. is nonsignificant.

Estimating the effective population size in windows across the genome should shed light on how fast selection and drift together cause allele frequencies to change. Using an estimator that relies on frequencies changing between any two time points (Jónás et al. 2016), we looked for differences in $N_{e}$ between chromosomes and treatments. Note that this approach uses data on any polymorphic sites and computes an estimate of variance $N_{e}$ which does not use any information from fixed loci. We chose this method as it provides accurate estimates in the presence of added sampling variance due to pool-seq and uneven sequencing depth. Previous results using molecular marker-based estimators, suggest that autosomal $N_{e}$ is similar between lines, ranging from 141.2 (s.d. 27.4) to 110.5 (s.d. 19.2) for M and E, respectively (Snook et al. 2009). Our results confirm that autosome-level estimates in M populations are within the predicted range (“Overall” in fig. 4) when considering AFCs between time points 1 and 5. Similarly, genome-wide $N_{e}$ is estimated at 149.3. In contrast, median autosomal $N_{e}$ in E lines is higher than expected at 154.7 (and 140.3 genome-wide; fig. 4). To investigate changes in $N_{e}$ throughout the experiment, we fit a generalized additive model (GAM) where we assigned “Replicate” as a random effect and “Time point” was defined as a predictor variable modeled with spline regression for each treatment (see Supplementary material online: Generalized Additive Model for $N_{e}$). We, therefore, investigated changes in $N_{e}$ through time for each treatment by examining the interaction effects of time across the two treatments. Results indicate that $N_{e}$ varies significantly through time in both E and M lines (Pvalue=0.001 and $<2\times 10^{-16}$ for effect of time point, respectively). Replicate has a marginally nonsignificant effect (Pvalue=0.067) but it should be noted that replicate-specific effects might not be captured with only four replicates. While these results are consistent with our experimental setup, the model explains 2.87% of the variation in $N_{e}$ estimates, suggesting that the great majority of variability in the data is either stochastic or determined by other unknown factors (see supplementary fig. S11, Supplementary Material online).

**Fig. 5.**
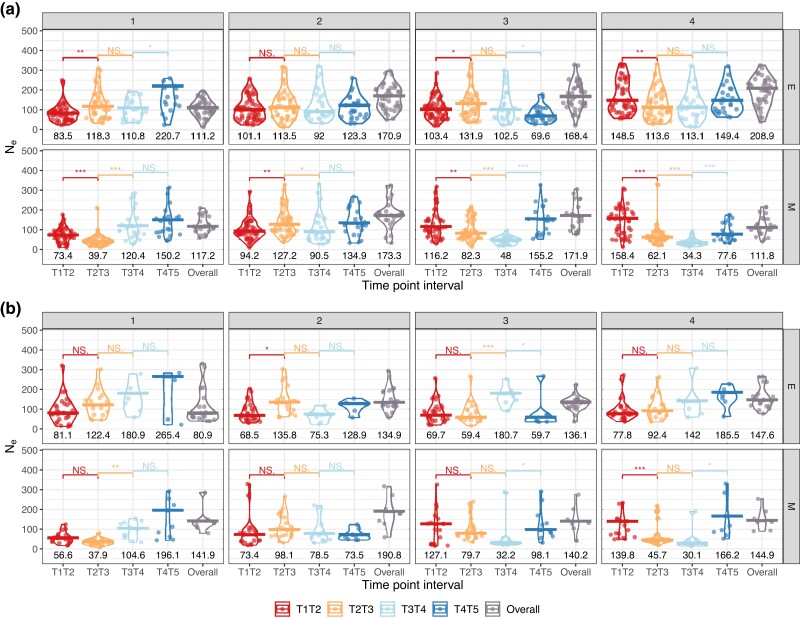
$N_{e}$ estimates at the (*a*) autosome- and (*b*) X chromosome-level for M and E lines at different time-point intervals. Violin plots and data points are included for each time-point interval and replicate per treatment. Outliers were removed. Medians are shown as bars and median estimates can be found at the bottom of each violin plot. These were calculated using all 2k SNP window estimates from the four experimental replicates. “Overall” corresponds to $N_{e}$ estimates based on AFCs between the first and last time point. Adjacent time-point intervals were compared using a Mann–Whitney *U* test. Significance level is indicated with *** for Pvalue<0.001, ** for Pvalue<0.01, * for Pvalue<0.05, and NS. is nonsignificant.

In E populations, genome-level $N_{e}$ drops most during the first 20–60 generations implying that selection is strongest then. This decrease is driven by reduced $N_{e}$ on the X chromosome (fig. 5). It reaches its lowest at T1–T2 for 3 out of 4 replicates, ranging from 68.5 to 81.1. $N_{e}$ starts to recover from time point 3 onwards reaching ≈122 on the autosomes and 147 on the X chromosome at the end of the experiment (“T4–T5” in figs. 4 and 5). Such a result is only possible because variance-$N_{e}$ is estimated from observed temporal shifts in allele frequencies. This is not caused by new mutations as we assume that adaptation occurs from standing genetic variation throughout this study. This same pattern is not found in M lines, where autosome-level $N_{e}$ estimates suffer a continuous and strong reduction until time point 4 (from 98 at generation ∼21–56 at generation ∼161), after which neutral levels are nearly recovered (fig. 4). The lowest overall estimates were found in M lines (e.g., replicate $3N_{eX}∼30.1$ and $N_{eA}∼34.3$; fig. 5) perhaps suggesting that genetic drift is strongest under M. Similar patterns are recovered when using only intergenic variants to estimate $N_{e}$ (supplementary table S5, Supplementary Material online).

We also observed that $N_{e}$ recovers towards the end of the experiment regardless of the treatment and whether we consider $N_{e}$ on the autosomes or the X chromosome (7 out of 8, and 6 out of 8 replicates increase in frequency during the last quarter of the experiment, respectively). This suggests that (i) selection acting in the first tens of generations of experimental evolution might be causing a substantial reduction in $N_{e}$ and (ii) selection during the last intervals of the experiment is less effective in altering allele frequencies, allowing $N_{e}$ to recover. In addition, the highest overall variance-$N_{e}$ estimates were observed through allele frequency changes between the first and last time point (“T1–T5”): on the X chromosome in M lines (3 out of 4 replicates) and at the autosome-level in E (3 out of 4 replicates).

Closer inspection of figure 5 shows that there is replicate-specific behavior. Replicate 1 is particularly worthy of note. First, there is a consistent pattern where $N_{e}$ estimates for both autosomes and the X chromosome are highest at “T4–T5” in the two treatments. In fact, replicate 1 shows the highest $N_{e}$ estimates throughout the experiment (E autosomes: 220.7; E X chromosome: 265.4). Interestingly, $N_{e}$ from changes between T1 and T5 is the nearest to Snook et al.’s (2009) census-based predictions for E lines—autosomes 111.2 versus expected 119.1, and X chromosome 80.9 versus expected 76.8. Our findings suggest that reduced $N_{e}$ in E lines, especially on the X chromosome, during the first quarter of the experiment—time points 1–2—might indicate a strong selective response if populations reach a new phenotypic optimum very swiftly. These results point to generally stronger selection on the X in comparison to the autosomes. In contrast, selection under M seems to act less effectively, causing low $N_{e}$ until the third quarter of the experiment. This pattern of delayed reduction in $N_{e}$ followed by a recovery to neutral levels is especially marked in autosomes.

Finally, we compared $N_{e}$ on the X chromosome to autosomal estimates by calculating $N_{eX}/N_{eA}$ ratios for each time-point interval (table 1). In E lines, $N_{eX}$ is greater than $N_{eA}$ during the last half of the experiment, that is, from time point 3 onwards. An implication of this is the possibility that the substantial increase in $N_{eX}$ may be caused by sex-specific associative overdominance maintaining slightly deleterious polymorphism in females. Furthermore, we compared our estimated ratios to those predicted by Snook et al. (2009): 0.75 and 0.65 for M and E lines, respectively, and found them consistently higher. For both treatments, we calculated 95% confidence intervals at each consecutive time-point interval, as well as for T1–T5 (i.e., “Overall”). We found that mean estimates were consistently greater than predicted. In particular, $N_{eX}/N_{eA}$ ratios were significantly greater than the expectation when considering $N_{e}$ estimates from T1 to T5 allele frequency changes—“Overall”—in both treatments as well as from T3 onwards in E lines (table 1).

**Table 1.** $N_{eX}$ versus $N_{eA}$ for M and E Lines at Different Time Point Intervals

$N_{eX}/N_{eA}$				
Time Interval	M		E	
	Mean	95% CI	Mean	95% CI
Overall	1.11	[0.77, 1.44]	0.76	[0.68, 0.83]
T1–T2	0.88	[0.64, 1.12]	0.71	[0.41, 1.01]
T2–T3	0.86	[0.67, 1.05]	0.87	[0.36, 1.39]
T3–T4	0.82	[0.66, 0.98]	1.37	[0.69, 2.04]
T4–T5	1.16	[−0.02, 2.33]	1.09	[0.81, 1.37]
Expectation	0.75		0.65	

Note.—Combined $N_{eX}/N_{eA}$ ratios for all replicates. “Overall” corresponds to $N_{e}$ estimates based on AFCs between the first and last time point. The percentage increase from the predicted ratios of 0.75 for M lines and 0.65 for E lines (as per estimated in Snook et al. 2009) are found in brackets.

### Estimating Selection

**Fig. 6.**
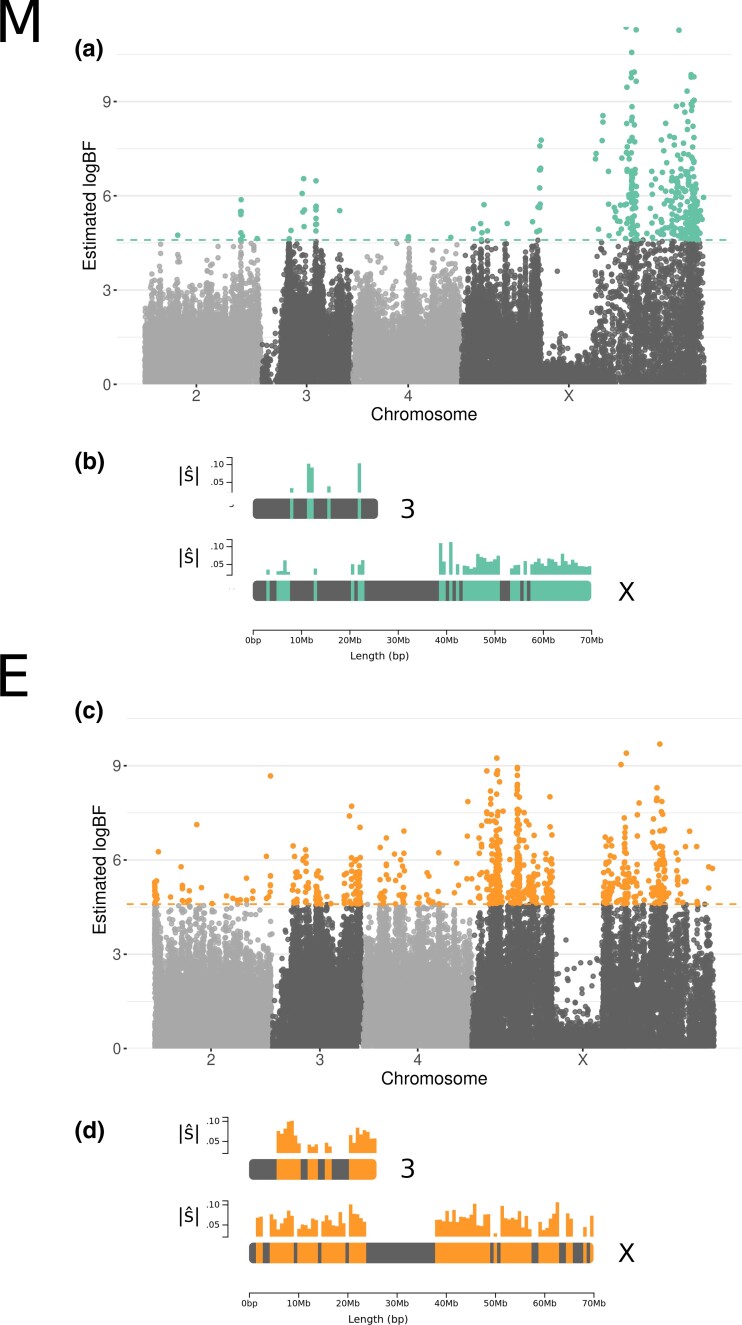
Genome scan for signatures of adaptation throughout the genome for M (top) and E (bottom) lines. (*a*) and (*c*) are Manhattan plots of Bait-ER (Barata et al. 2023) log BF for each allele frequency trajectory. Statistically significant SNPs are colored in green (M, top) or orange (E, bottom). Dashed lines correspond to a threshold of log(99)≈4.6. (*b*) and (*d*) are diagrams of chromosomes 3 (top) and X (bottom) that illustrate which regions of each chromosome harbored the most number of significant hits. Average estimated selection coefficients ($\left|\hat{s}\right|$) for each interval can be found above each diagram as a bar plot. Data excludes chromosome 5.

We performed a genome scan of the time-series data across all time points using Bait-ER (Barata et al. 2023). We have chosen this method as a genome scanner for selective sweep-like trajectories. It accounts for sampling variance while searching for consistency across replicates. Bait-ER has been shown to perform well in small population experiments with few replicates. The signal of selection is substantially higher in E versus M lines (fig. 6*a* and *c*), which suggests that selection is indeed stronger under E. In total, 350 (0.9% of all sites in the time series) and 770 (1.5%) SNPs were statistically significant (at a threshold of log(99)) for M and E lines, respectively. If considering the first 3 time points alone, M lines had 570 (0.6%) significant SNPs whilst E had 1591 (1.5%). Regardless of whether you consider the complete time series or a shorter data set with the first 3 time points only, E populations have a similar percentage of sites—1.5%—that are considered to be under selection. They consistently show approximately double the number of loci with evidence of selection than the M lines. Moreover, the location of selected trajectories is similar to the full time series only the selection signal is more widespread along chromosome 3 in E lines (supplementary fig. S13, Supplementary Material online).

A comparison of candidate allele frequency trajectories revealed only small differences between the two treatments. If we consider 5 time-point trajectories, alleles with the highest log BF (> Q3 in supplementary figs. S14 and S15, Supplementary Material online) approach fixation within the first 60–115 generations (time points 2 and 3). By time point 4, that is, the last quarter of the experiment, nearly all selected sites are fixed, which is especially noticeable in E lines across all log BF quantiles. Our 3 time-point trajectory analysis reveals that most alleles were fixed or near fixation at time point 3 which is consistent with 5 time-point trajectory results (supplementary figs. S16 and S17, Supplementary Material online). However, E lines show more polymorphism in comparison to M populations especially for log BFs that fell below the median value. Under M, we observed that replicate 3 differed from the other replicates showing a substantial proportion of polymorphic sites by the end of the first half of the experiment. If we now turn to the distribution of starting allele frequencies for those sites targeted by our genome scan, we can see that starting frequencies are slightly more shifted towards intermediate values in E versus M lines but this difference varies between the replicates (supplementary fig. S12, Supplementary Material online). These folded starting frequency distributions also suggest that candidate alleles fall outside the lowest frequency intervals which contain the boundary frequency states.

When comparing the different chromosomes, it is clear that there are far more significant peaks along the X chromosome in comparison to autosomes in both treatments (M: 322; E:563). Of those 322 top X candidates in M lines, 309 (96%) were located in intergenic regions (vs. 50.8% in E). This is a surprising result given that half of the variants called on the X can be found within intergenic regions. Significant trajectories on chromosomes 2 and 4 were never seen for more than 60 SNPs (2: 9 and 43; 4: 3 and 54; for M and E, respectively). In addition, evidence for selection on the third chromosome is also markedly elevated in E populations where there are 110 significant SNPs versus only 16 in M (fig. 6*a*–*d*). Taken together, these results suggest that whilst selection is stronger under E, the X chromosome is also responsible for adaptation to a strict M regime.

**Fig. 7.**
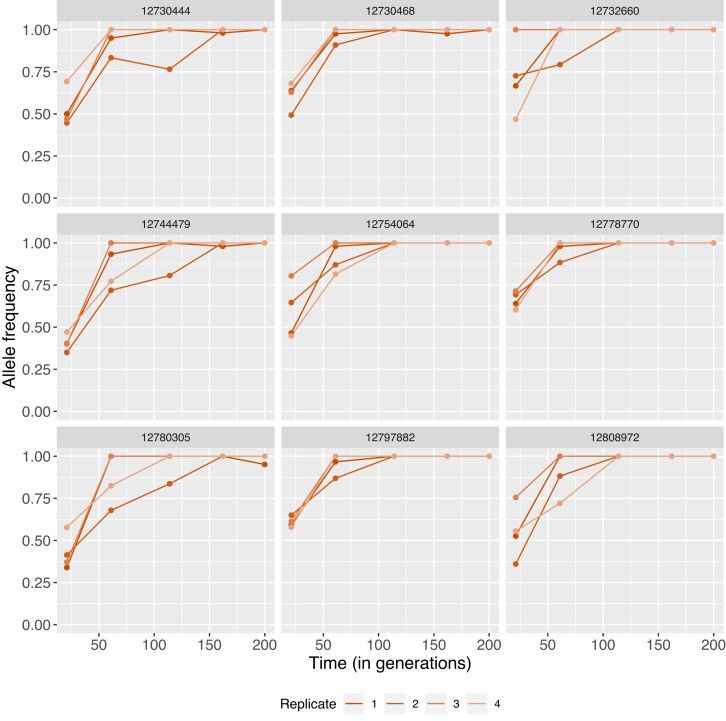
Nine (out of 11) allele frequency trajectories of significant SNPs located on a single gene coding for neuromusculin. Each replicate is colored differently as per bottom legend. Individual variant coordinates can be found at the top of each graph.

In E lines, 417 (54.2%) of those statistically significant variants were found within genes, whereas only 35 SNPs (10%) were mapped within genes in M lines. This difference is striking given that approximately the same number of significant variants were located in intergenic regions (E: 353; M: 315). In addition, we observe that there are more candidate SNPs in intergenic regions than expected by chance (E: chi-squared 59.993, d.f. 1, $P\;\text{value}<9.521\times 10^{-15}$; M: chi-squared 265.02, d.f. 1, $P\;\text{value}<2.2\times 10^{-16}$). We also found that 141 (out of 353 intergenic SNPs; i.e., 40%) and 13 (out of 315 intergenic SNPs; i.e., 4%) were located within 15 kbp up- or downstream of the closest gene for E and M lines, respectively. A large proportion of the E line top variants that were found in genes locate to the X chromosome (426, 72.3%). Fourteen genes in E populations have 5 or more significant SNPs (up to 23; supplementary table S10, Supplementary Material online). Selection coefficients for individual trajectories as estimated with Bait-ER ranged from 0.03 to 0.11. Genes with the highest number of top SNPs were found on the X and third chromosomes. Of these, the second gene with the most top variants (11) codes for hemicentin-1 (NCBI: 4813557; FlyBase: FBgn0076932) with an ortholog in *Drosophila melanogaster*—neuromusculin which is a protein that is expressed in the muscle system as well as the peripheral nervous system. Allele frequency trajectories for these top SNPs typically start at relatively high frequency and fix within two or three time points (fig. 7).

Wiberg et al. (2021) previously identified 480 variants as having a significant allele frequency differences between M and E replicates at time point 3. We determined which genes these top SNPs were located in as well as any genes in the vicinity of intergenic top SNPs. We then compared these genes with those found significant in our genome scan. There were 21 genes in common between the two studies (supplementary table S9, Supplementary Material online) suggesting the majority of genes showing differentiation between treatments differ from those with sweep-like frequency trajectories. However, under polyandry, more genes with significant hits were found in common with Wiberg et al. (2021) on average than expected by chance (chi-squared 18.343, d.f. 1, $P\;\text{value}<1.845\times 10^{-05}$). These include genes involved in neural and muscle development, as well as other biological regulation processes.

## Discussion

### Predictions for the Genomic Response to Sexual Selection

Sexual selection can cause substantial divergence between populations and is thought to be involved in speciation (Seehausen et al. 2014; Janicke et al. 2018). It has been repeatedly implicated in altered ratios of genetic diversity between sex chromosomes and autosomes (e.g., Corl and Ellegren 2012). Here, we used an E&R experimental design in *D. pseudoobscura* to help elucidate the process of adaptation when the strength of sexual selection is altered in the short-term. We altered the intensity of sexual selection by reducing it in monogamous populations (M) or elevating it in a polyandrous regime (E). Signals of selection were strongest in E populations where sexual selection is elevated. This response is accompanied by a reduction in nucleotide diversity and more alleles becoming fixed as time progresses. In addition, $N_{e}$ estimates suffer a reduction as populations adapt, but recover towards neutral levels.

While M lines should exhibit relaxed selection since competition for mates is eliminated, E lines are likely to be subject to elevated selection. Increasing the number of males a single female is housed with should cause sexual selection to be stronger. The E regime thus results from the observation that *D. pseudoobscura* are naturally polyandrous and each female has been found to mate with 2–3 males within its lifetime (Dobzhansky and Pavlovsky 1967). Therefore, housing a female with six males should increase intrasexual competition. One would predict that increased promiscuity would facilitate the evolution of traits involved in mating or fertilization success and perhaps prezygotic isolation mechanisms between the two treatments. Our *D. pseudoobscura* populations show a lack of assortative mating either between treatments or within lines (Debelle et al. 2016). A possible explanation for this might be that the effects of elevated male-male competition have overcome the coevolution of female preference in this experiment. This observation could help generate expectations regarding some of the genomic response to selection. In a system where female preference is overshadowed by competition amongst males, sexual conflict is likely to increase in E lines. On the other hand, competition for mates is eliminated under M. This could allow for sexual conflict to be reduced, causing potentially sexually antagonistic variation to decrease through the fixation of polymorphic sites.

Adaptation to an altered mating system will shape patterns of genetic variation in somewhat unpredictable ways. Understanding how AFCs occur within given haplotype structures is instrumental to finding putative targets of selection. Is the signal of adaptation to sexual selection consistent throughout the genome and across time? Our approach to understanding the adaptive process relied on taking snapshots of the replicates at several time points throughout the 200 generation experiment. These snapshots were allele frequencies estimated from a pool-seq data set of each of the four replicate populations. We ensured that only high quality SNPs are present in both the full time series and the two time point data sets. These SNPs were identified by keeping the variants that were consistently called by two separate variant callers and previously mapped with two different read mapping approaches. In particular, selection scan results are based on a time series that is comprised of SNPs that were polymorphic at the first time sampling point. This causes our results to be focused on polymorphisms with the most adaptive potential, since most would have higher potential to overcome the counteracting effects of drift over the first 20 generations of selection. In small populations such as these, drift will cause alleles to shift such that most low frequency polymorphisms will be lost within a few generations.

### Strong Selection under Both Treatments

Taken together, our results support the hypothesis that strong sexual selection in E lines causes a substantial adaptive response. Not only did alleles become fixed more promptly in E populations (fig. 1), but also nucleotide diversity was depleted faster (fig. 2). In addition, our genome scan showed more than double the number of target candidates in E versus M. These top SNPs were found mostly across the X and the third chromosomes (fig. 6*a*–*d*). These results are consistent with the findings by Wiberg et al. (2021) where SNPs showing significantly consistent allele frequency differences at the third quarter of the experiment between E and M clustered along chromosomes 3 and X. The selection signatures we find are more pervasive in comparison to Wiberg et al.’s “islands” of differentiation. This is perhaps suggesting that LD has a substantial impact in our genome scan. In addition, effective population size estimates are consistent with a swift response to selection from the start of the experiment, especially in E. $N_{e}$ estimated from AFCs between time points 1 and 2 indicate that E lines suffer a more drastic reduction from the onset of selection.

Wiberg et al. (2021) found a cluster of top SNPs on chromosome 3 that showed significant differentiation between M and E lines. High levels of nucleotide diversity observed at the start of the experiment (supplementary fig. S8, Supplementary Material online) make chromosome 3 a good candidate for harboring selection targets. This would facilitate adaptation due to increased fitness variance amongst individuals in the population. The region at the end of the chromosome was identified by Wiberg et al. as also showing a steep rate of decay in LD. This suggests that this peak region exhibits high recombination which is unexpected given that telomeres are typically low recombination regions. Our study confirms these results. There is evidence for positive directional selection within this region. Interestingly, the signal seems to be caused solely by directional selection in E lines. Increased recombination at the end of chromosome 3 relative to neighboring areas could have contributed to the slightly elevated nucleotide diversity (supplementary fig. S8, Supplementary Material online). This region does not overlap with known inversion breakpoints on chromosome 3 (Wallace et al. 2011). Inversions have been identified on the X but not fully mapped (Ortiz-Barrientos et al. 2006), so it is possible that structural variants on the X could impact our results.

At the end of the first half of the experiment—between time points 2 and 3—E populations showed far more fixations than M lines (fig. 1). We hypothesized that this could result in a more marked response to selection in E which could, in turn, manifest as a reduction in $N_{e}$ within that time interval. Interestingly, the pattern is reversed when comparing $N_{e}$ between the two treatments: M lines have a much lower overall $N_{e}$ on average than E. This result is statistically significant (M vs. E at T2–T3 Mann–Whitney *U* test $P\;\text{value}=8\times 10^{-13}$). This could mean that, despite more overall fixed loci, variance-$N_{e}$ at the end of this first half indicates a more substantial reduction amongst M populations. A pattern such as this might be caused by one of two things. First, drift could be stronger in M overall resulting in drift variance that is picked up by the $N_{e}$ estimator. Other monogamous regimes have been shown to result in lower overall $N_{e}$ which augments the extent of drift (Wigby and Chapman 2004). The prediction that $N_{e}$ differs between the treatments was tested previously in our lines and found not to be significant (Pvalue=0.52) (Snook et al. 2009). Secondly, our estimates could be biased if selection affects most AFCs. This effect should dissipate if one would estimate $N_{e}$ with sites that are evolving neutrally similarly to Snook et al.’s molecular marker-based approach. We tried to overcome this by computing $N_{e}$ using intergenic SNPs alone. General trends remained unchanged with similar median $N_{e}$ for M and E between T2 and T3 as that using the complete data set (M: 68.2 and E: 111.9; supplementary table S5, Supplementary Material online).

As costs of promiscuity arise from different mating frequency optima between the sexes, M lines could show signs of evolution under reduced levels of sexual conflict. This could, in turn, result in a weaker selective sweep signal throughout the genome. Monogamous populations do exhibit less response to selection with fewer significant hits across the genome. This finding can be evidence for relaxed sexual selection due to mate competition being eliminated. However, the genomic location of such significant hits is quite telling—90% of candidate sites were located in intergenic regions in M lines. If changes in gene expression are involved in the response to sexual conflict (Hollis et al. 2014; Veltsos et al. 2017), mutations in regulatory regions could facilitate this process. It is, thus, possible that those regions showing selection signatures in M lines may have harbored a substantial portion of the initial sexually antagonistic variation. We predict that the point at which female and male phenotyic optima converged would be accompanied by a substantial reduction in variance-$N_{e}$. In other words, we expect the lowest $N_{e}$ estimates to correspond to the time-point interval where selection is at its strongest. Interestingly, median $N_{e}$ in M throughout the experiment is severely reduced until time point 4 to values lower than those found in E. An accelerated rate of genetic drift due to M cannot be ruled out here. However, this could, alternatively, be evidence of a delayed response where new phenotypic optima are reached towards the last quarter of the experiment.

### Adaptation on the X Chromosome

Most of the adaptive signal is found on the X chromosome, which agrees with earlier studies on the genetic basis of both intra- and intersexual conflict (Gibson et al. 2002; Connallon and Jakubowski 2009; Mank and Ellegren 2009). These suggest that X-linkage is likely to facilitate the response to sexual selection through the accumulation of sexually antagonistic variants. Our genome scan shows a clear response that is widespread across the X in E lines, with 72% of candidate sites found within genes. This is consistent with a faster-X effect where protein-coding genes on the X show rapid evolution (Charlesworth et al. 1987). However, $N_{eX}/N_{eA}$ ratios are consistently greater than estimates predicted under neutrality which contradicts the evidence for a faster-X effect. In fact, this suggests that reduced recombination on chromosome X could favor diversity to be maintained along extended haplotypes. This could be introducing a lack of power to detect a faster-X effect using $N_{e}$ as a proxy for diversity. Determining the extent of linkage disequilibrium in this experiment is difficult as there is no data on the haplotypes present in the founder populations in our pool-seq approach. Nevertheless, signatures of adaptation emerging from examining the X chromosome are still compelling.

Our results suggest that the predicted 3/4 reduction in $N_{e}$ on the X chromosome under M is not prevalent throughout the experiment. $N_{eX}$ is just as high as on the autosomes in M lines regardless of the time point interval in question. A similar trend is observed in E lines where $N_{eX}$ is even found to be significantly greater than $N_{e}$ estimates on the autosomes during the second half of the experiment. This is a striking result as theoretical studies predict a lower $N_{eX}$ (Betancourt et al. 2004; Pool and Nielsen 2008), especially under polyandry. Higher $N_{eX}$ indicates that genetic diversity on the X is similar to that found in the autosomes. Accordingly, the elevated Tajima’s D we observe throughout the genome is also consistent with an excess of heterozygosity. One possible explanation might be that balancing selection could be maintaining higher levels of polymorphism which could increase estimates of variance-$N_{eX}$. In such cases, pervasive balancing selection might arise especially if a large proportion of this variation is antagonistic between the sexes, which would favor heterozygotes. Alternatively, any excess heterozygosity may be caused by large-scale sex-specific associative overdominance where there is an apparent heterozygote advantage (or pseudo-overdominance) at effectively neutral loci due to linked selection in females. This results from linkage desequilibrium between a neutral polymorphic locus and other loci under balancing selection (Ohta and Kimura 1970). Purifying selection against recessive deleterious variants can also cause associative overdominance (Ohta 1971).

As far as the X chromosome is concerned, the response found on the two chromosome arms differs between the treatments. M lines had a more pronounced response towards the distal end of the chromosome, whereas E lines’ signal was more evenly distributed along the chromosome. This suggests that the genetic basis of adaptation to either elevated or relaxed sexual selection on the X is distinct. This pattern would not be detected in most studies which simply compare divergence in allele frequencies between any two lines. The distal end of the X chromosome (chromosome arm XR in previous *D. pseudoobscura* assemblies) is equivalent to Muller element D in the *D. melanogaster* genome. This chromosome arm is known to have fused with the ancestral X chromosome to form the “neo-X.” This could indicate that chromosome arm XR harbors genes that are key to the response to increased mate competition. Moreover, both treatments exhibited a marked valley of signals of selection (fig. 6*a*–*d*) in the center of the X. This was coupled with a positive and elevated Tajima’s D within the same region (fig. 3). Such a pattern suggests that, in spite of evidence for positive selection on both chromosome arms, the centromere region could be under balancing selection. This could perhaps be the result of sex-specific allele differentiation on the X between the two sexes. The center of the X also contains some of the highest coverage regions across the genome (supplementary fig. S18, Supplementary Material online). This indicates that it might be a highly repetitive portion of the chromosome. Phased data from long read sequencing technology would be necessary to resolve this issue.

### Conclusion

In summary, we showed that the response to an altered mating system in populations of *D. pseudoobscura* is found mostly on the X chromosome and also on chromosome 3. This is consistent with previous work that focused on comparing E and M lines at time point 4 in our analysis (Wiberg et al. 2021). Selection signals are is strongest in E lines when mate competition is strongest due to E. Such a pattern indicates that most AFCs observed were in fact caused by elevated sexual selection and not solely adaptation to lab conditions. Our study showed the power of investigating allele frequency trajectories and their usefulness when estimating selection parameters and the effective population size.

Overall, populations recovered to neutral levels of $N_{e}$ towards the middle or end of the experiment for E and M lines, respectively. Here, variance in AFC match expectations from a neutral drift model, which might indicate the end of an initial strong selection phase. It is possible that phenotypic optima are reached at that point and allele frequencies might plateau. In other words, directional selection becomes less effective towards the end of the experiment as populations reach new phenotypic optima. Selection coefficients are reduced as polymorphism is eliminated and other modes of selection may arise. Stabilizing selection will prevent trait means from moving away from the new optima. Any remaining genetic variation at the end of the experiment resulted in a proportion of variance in AFC that was similar to that expected from a neutral drift model. Again, balancing selection may now be present and act to maintain some genetic diversity. Our results are consistent with a general picture of increased sexual antagonism in the E lines but an elimination, requiring changes in expression, in the M lines.

## Material and Methods

### Experimental Setup

The experiment was established in 2002 and lasted for approximately 200 generations (Snook et al. 2005). The ancestral population was established from 50 wild-caught females collected in Arizona, USA. The selection experiment was set up after four generations of “common-garden” laboratory evolution. Each of the two treatment lines (M and E) was replicated four times. For each selection regime, recently eclosed offspring were collected and combined given the appropriate sex ratio at every generation. All experimental populations were kept with standard food media and added live yeast at 22${}^{\circ }$C on a 12-L:12-D light cycle. For a more detailed description, see Snook et al. (2005) and Crudgington et al. (2005).

Both selection regimes were established based on the observation that *D. pseudoobscura* females are assumed to carry sperm from at least two males at any given time in the wild (Cobbs 1977). Therefore, for each E treatment group, two M line groups were established. For each replicate population, 80 and 40 groups of flies were established at each generation in M and E treatment lines, respectively. Family and population sizes were chosen according to census-based $N_{e}$ estimates to ensure that both treatment did not vary in their effective population size. These expectations were tested and found to hold after approx. 26 generations of experimental evolution (Snook et al. 2009). Consequently, we expect no differences in the potential for adaptation between treatments due to reduced effective population size.

### Sequencing

The time-series data set consists of five time points and it includes all four replicates for each selection regime. These time points are fairly evenly distributed throughout the study. The experimental setup was such that the replicates were established in a staggered fashion. Therefore, fly sampling did not occur at the same for all replicate populations: time point 1 was sampled at generations 21 and 22, time point 2 between 59 and 63, time point 3 between 112 and 116, time point 4 ranged between 160 and 164, and time point 5 at generation 200. For more details on the generation at which each replicate was sampled and, thus, sequenced, see supplementary table S1, Supplementary Material online. Samples at time point 4 were sequenced as part of Wiberg et al. (2021).

All fly samples were stored at −80${}^{\circ }$C immediately after collection in the Department of Animal and Plant Sciences at the University of Sheffield. The samples were then collected and kept at −80${}^{\circ }$C storage in the Centre for Biological Diversity at the University of St Andrews up until DNA extraction.

For each DNA sample, 40 female flies were pooled from the frozen stocks. Females were sexed and collected approximately at time of emergence, thus, we assume these to be virgin. DNA extraction was performed using a DNeasy Blood & Tissue Kit (QIAGEN) for 20 individuals. Firstly, flies were homogenized at room temperature using a Bullet Blender homogeniser with zirconium beads. After adding Proteinase K, all samples were left to incubate overnight at 56${}^{\circ }$C. The step which involves adding buffer AW1 was repeated, and the elution with buffer AE (150 μL) was also repeated to maximize DNA yield. At the end of the extraction protocol, the two 20-female samples were combined to make up a pool of DNA and stored at −20${}^{\circ }$C. DNA concentration increased as time progresses, with time point 1 at 34.9 ng/μL, time point 2 at 45.1 ng/μL, time point 3 at 45.4 ng/μL, and time point 5 at 50.8 ng/μL. This is consistent with more recent DNA samples being better preserved.

DNA sequencing was carried out at Novogene (Hong Kong) using an Illumina HiSeq X Ten platform. The library preparation protocol resulted in a 350 base-pairs (bp) insert DNA library. For each sample, there is a set of raw paired-end reads all 150 bp long.

### Read Mapping

Raw reads were filtered and trimmed using Trimmomatic (version 0.38, Bolger et al. 2014). After trimming, time points 1, 2, 3, and 5 had an average read length of 148 (min=36, max=150), whilst time point 4 had shorter reads with an average length of 97.3 (min=36, max=100). Trimmed reads were then mapped to both the complete *Drosophila pseudoobscura* genome assembly Dpse_4.0 (FlyBase, GenBank accession GCA_000001765.3, June 2018) and the X chromosome sequence of the UCBerk_Dpse_1.0 assembly (UC Berkeley, GenBank accession GCA_004329205.1, March 2019). The former reference was assembled with Illumina (150×) and PacBio (70×) reads, and the latter consists of Oxford Nanopore MinION (40×) long reads.

All paired-end reads were mapped separately using two mappers: bwa mem (version 0.7.17, default parameters, Li 2013) and novoalign (version 4.00.Pre-20190624, Novocraft Technologies, http://novocraft.com/). Regardless of which mapper was used, over 98% of reads were mapped successfully to the reference genome (see supplementary table S2, Supplementary Material online for more details). The SAM files produced by the two mappers were re-aligned to around indels using GATK (Genome Analysis Tool Kit, version 3.8.1, Van der Auwera and O’Connor 2020).

### Variant Calling and Filtering

Variants were then called with both bcftools (mpileup and call functions, version 1.9, Li 2011; Danecek et al. 2021) and freebayes (version 1.3.3, Garrison and Marth 2012) (see supplementary table S3, Supplementary Material online for details on variants called by both callers). Variants called by bcftools were filtered according to the following criteria suggested by Kofler et al. (2016):

1. Minimum mapping quality of 40;
2. Minimum base quality of 30;
3. Minimum allele count of 1/F at the first time point, where *F* is the total number of founder haplotypes or the sample size, that is, MAF=1/40=0.025;
4. Remove sites not called by FreeBayes.

In addition, we have filtered out those variants not present in both the bwa mem and novoalign mapped data sets. Such a two-mapper approach to producing pool-seq data is rather conservative and was preferred to ensure good quality data sets. Genome-wide, novoalign alignments led to fewer called mean SNPs genome-wide: 2,309,226 with bwa mem versus 2,194,721 with novoalign for M lines, and 2,281,034 with bwa mem versus 2,167,341 with novoalign for E populations. A similar trend is observed in the X chromosome alignment (bwa mem 862,881 and 841,935 vs. novoalign 807,689 and 787,561 for M and E, respectively). Fewer variants were called as time progressed. On average, a little over a third of variants that were called on autosomes were intergenic (chromosome 2: 34.1%; chromosome 3: 34.1%; chromosome 4: 38.6%). A higher percentage was observed on the X chromosome where 47.5% of variants were intergenic. We also considered strand bias (quantity that measures whether a SNP is just as likely to be found on the forward and the reverse strand; it is calculated as $\left|f_{\;\mathrm{forward}}-0.5\right|$, where $f_{\;\mathrm{forward}}$ is the proportion of reads that were mapped to the forward strand at any given site; supplementary fig. S4, Supplementary Material online) and overall coverage for each analyzed locus as a measure of quality (supplementary fig. S5, Supplementary Material online). After filtering for mapping and base calling quality, as well as retaining any variants called by bcftools and Freebayes in the two alignments produced with bwa mem and novoalign, median sequencing depth is 45×. For this reason, and for our choice of methods for downstream analyses which account for sampling variance due to uneven coverage, we decided not to filter variants based on coverage any further. Finally, we have only considered biallelic sites and of those only the ones that were found to be polymorphic at the first sampled time point were used for further genome scan analyses. This allowed us to describe AFCs throughout the experiment. In TP1 samples, approx. 131M sites were invariant reference alleles. Of these, approx. 126M were also present in TP2, which is to say that 3.45% of all invariant reference sites were not called in TP2. Our filtering criteria for TP1 samples removed, on average, 0.09% (116K sites) of fixed reference alleles which are, in turn, heterozygous at TP2. Despite losing these potentially informative sites, we argue that having allele frequencies from generation 60 onward would result in a substantial loss of power to detect selection on these trajectories. The total number of SNPs remaining after quality filtering as well as those called in both bwa mem and novoalign alignments for the whole-genome and the X chromosome assemblies can be found in supplementary table S3, Supplementary Material online. After filtering, SNPs present across all replicates between the five time points (or the first three) were considered for further selection inference. We analyzed 38,065 and 51,339 full 5 time point trajectories in M and E lines, respectively. For $N_{e}$ estimation, those variants present between any two time points were considered. More details on how these were distributed across time points and chromosomes can be found in supplementary table S4, Supplementary Material online and supplementary fig. S6, Supplementary Material online.

To investigate how quickly alleles were fixing throughout the experiment, we calculated experimental fixation rates that correspond to the number of SNPs that become fixed from one time point to the next. Experimental fixation rates were calculated as follows:


$$\mathrm{Fixation}\;\mathrm{rate}=\frac{\;f_{T_{n}}-f_{T_{n-1}}}{t_{n}}$$

where $f_{T_{n}}$ and $f_{T_{n-1}}$ are the number of fixed sites at time point *n* and time point n−1, and $t_{n}$ is the total number of trajectories being analyzed.

### Genetic Diversity

We used Grenedalf to calculate estimates of nucleotide diversity and Tajima’s D (Czech and Exposito-Alonso 2021). Grenedalf computes measures of diversity corrected for pool-seq. This includes $\theta_{\pi }$, hereinafter referred to as π. The program follows the approach implemented in PoPoolation (Kofler et al. 2011) and PoPoolation2 (Kofler, Pandey, et al. 2011) that accounts for any bias introduced by sampling and sequencing error. Absolute π is the sum of estimates for all SNPs within a given window, and relative π the average per window. We used sync format files, which include all replicates and time points, as input. We considered sample sizes of 40 individuals and computed π as well as Tajima’s D for each replicate at each time point in windows of 250 kbp with a 25 kbp overlap.

### Selection Inference and $N_{e}$ Estimation

For estimating the effective population size, $N_{e}$, we used a moment-based estimator (Jónás et al. 2016) implemented in the R package poolSeq (Taus et al. 2017). The estimator uses temporal data to investigate any AFCs. It accounts for the effect of pool-seq by introducing variance from two sampling events: first, when individuals are sampled from an experimental population for sequencing, and second, due to uneven coverage throughout the genome. The approach models drift variance to obtain a temporal estimator for $N_{e}$ (also referred to as variance-$N_{e}$). Estimates were computed for 2k SNP windows with a 10% overlap assuming that populations were sampled according to Jónás et al.’s plan II, where sampling takes place before reproduction and sampled individuals’ genomes do not contribute to the next generation. Chromosome-, autosome- and genome-level median estimates are computed for each replicate separately or by combining replicate data. Adjacent time-point intervals and treatments were compared independently using a Mann–Whitney *U* test computed with the R package *ggsignif* (Ahlmann-Eltze and Patil 2021), and a GAM was used to investigate changes in $N_{e}$ throughout the experiment with R package *mgcv* (Wood 2011).

Finally, we investigated potential targets of selection using a Bayesian genome scan on the time series—Bait-ER (Barata et al. 2023). It was designed for E&R experiments as it accounts for added binomial, or beta-binomial, sampling noise from pool-seq. Bait-ER models the evolution of an allele using a Moran model with overlapping generations. It estimates parameters of selection, namely selection coefficients (σ), whilst also testing each allele frequency trajectory for selection. Bait-ER searches for allele frequency trajectories that are consistently shaped like a selective sweep across replicates, and incorporates that information into the likelihood calculation. The program outputs a Bayes Factor (log BF) per site, which is a ratio of the likelihoods of two alternative models: one where genetic drift is the main driver of allele frequency changes, and another where there is positive selection favoring a particular allele. Similar to Grenedalf, both Bait-ER and poolSeq take sync files as input. Diagrams of chromosomal regions showing significant hits (fig. 6*b* and *d*) were produced using the chromoMap R package (Anand and Rodriguez Lopez 2022).

### Gene Feature Analysis

In order to obtain a complete annotation of gene features for our time-series data set, we used NCBI’s Remap tool (www.ncbi.nlm.nih.gov/genome/tools/remap). This tools allowed us to perform coordinate remapping between the latest annotated reference genome uploaded to NCBI’s repository (University of California, Irvine, Dpse_MV25, accession number GCA_009870125.2) and the two assemblies described in “Sequencing.” The software outputs gff3 format annotation files which we converted to bed format using BEDOPS’ (Neph et al. 2012) gff2bed tool.

## Supplementary Material

evad113_Supplementary_Data

## Data Availability

The genomic data generated in this article can be found in the NCBI’s Sequence Read Archive (SRA, https://www.ncbi.nlm.nih.gov/sra). Raw reads were deposited under the BioProject ID PRJNA808747. All scripts used for read mapping, variant calling and filtering, estimating $N_{e}$ across the genome, and inferring selection from standing genetic variation can be found here: https://github.com/carolbarata/dpseudo-n-beyond.
