# Supplementary material for: Selection on the Fly: Short-Term Adaptation to an Altered Sexual Selection Regime in Drosophila pseudoobscura
Source: Genome Biol Evol. 2023 Jun 21;15(7):evad113. doi: 10.1093/gbe/evad113 (PMC10319773; doi:10.1093/gbe/evad113)
Supplement: evad113_Supplementary_Data [file evad113_supplementary_data.pdf]

Supplementary information

Mapping statistics

	Generation				
Replicate	TP 1	TP 2	TP 3	TP 4	TP 5
M/E 1	21	63	116	164	200
M/E 2	21	62	115	163	200
M/E 3	21	61	114	160	200
M/E 4	22	59	112	160	200

Table S1: **Description of which generations were sampled at each time point.**

Treatment	Mapper	Average no.	Average %	Minimum no. (%)	Maximum no. (%)	Assembly
M	bwa	48,315,592	99.0	37,798,247 (98.6)	62,704,196 (98.8)	Whole genome
M	novoalign	48,629,415	99.2	38,092,806 (98.9)	61,869,582 (99.2)	Whole genome
E	bwa	52,169,450	99.0	43,105,534 (98.8)	65,961,589 (99.2)	Whole genome
E	novoalign	52,568,742	99.2	43,479,141 (99.1)	66,521,236 (99.3)	Whole genome
M	bwa	22,269,730	98.4	17,630,965 (98.1)	28,450,846 (98.3)	X chromosome
M	novoalign	22,712,923	98.8	17,883,361 (98.6)	29,118,289 (98.8)	X chromosome
E	bwa	24,014,102	98.4	19,722,067 (98.3)	29,666,606 (98.8)	X chromosome
E	novoalign	24,494,392	98.9	20,185,805 (98.8)	30,279,204 (99.0)	X chromosome

Table S2: **Mapping statistics for both mappers.**

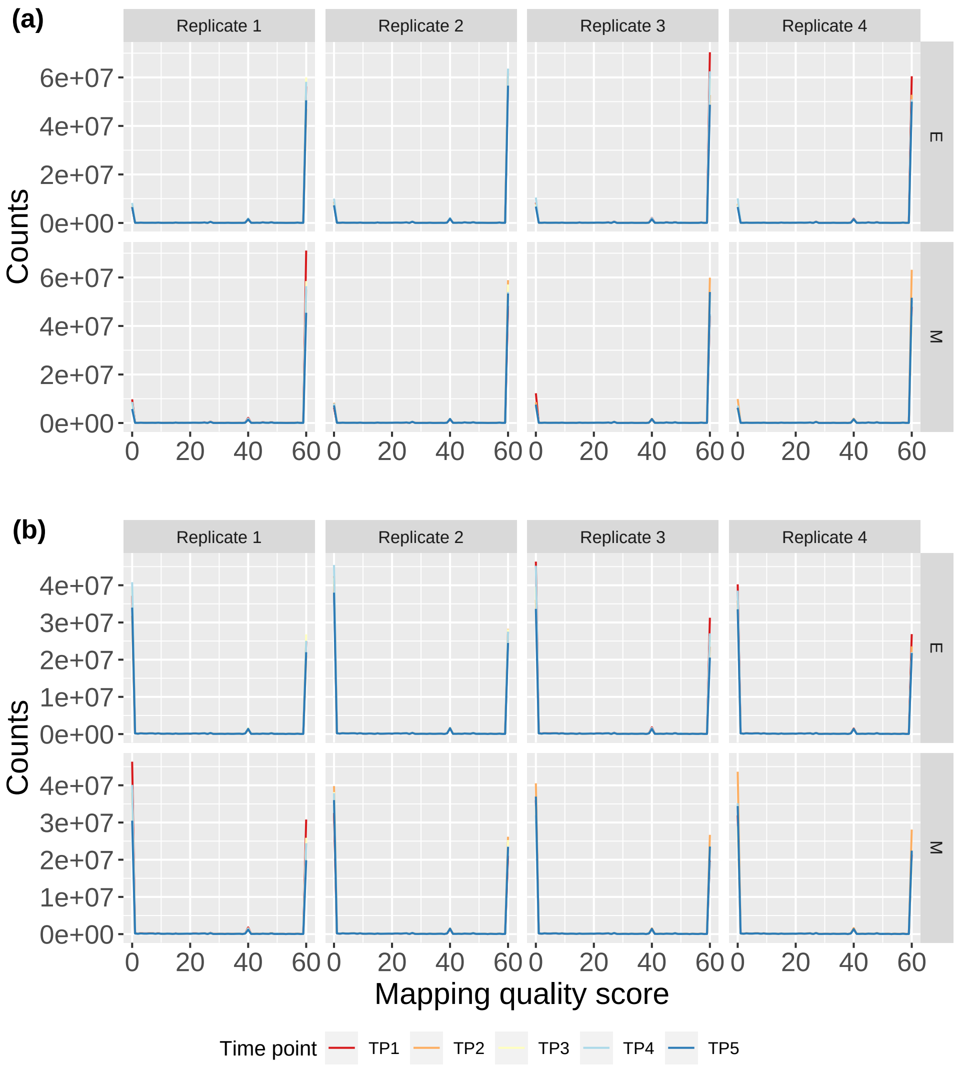

Figure S1: **Mapping quality score distribution at the genome (a) and X chromosome (b) level assemblies.**

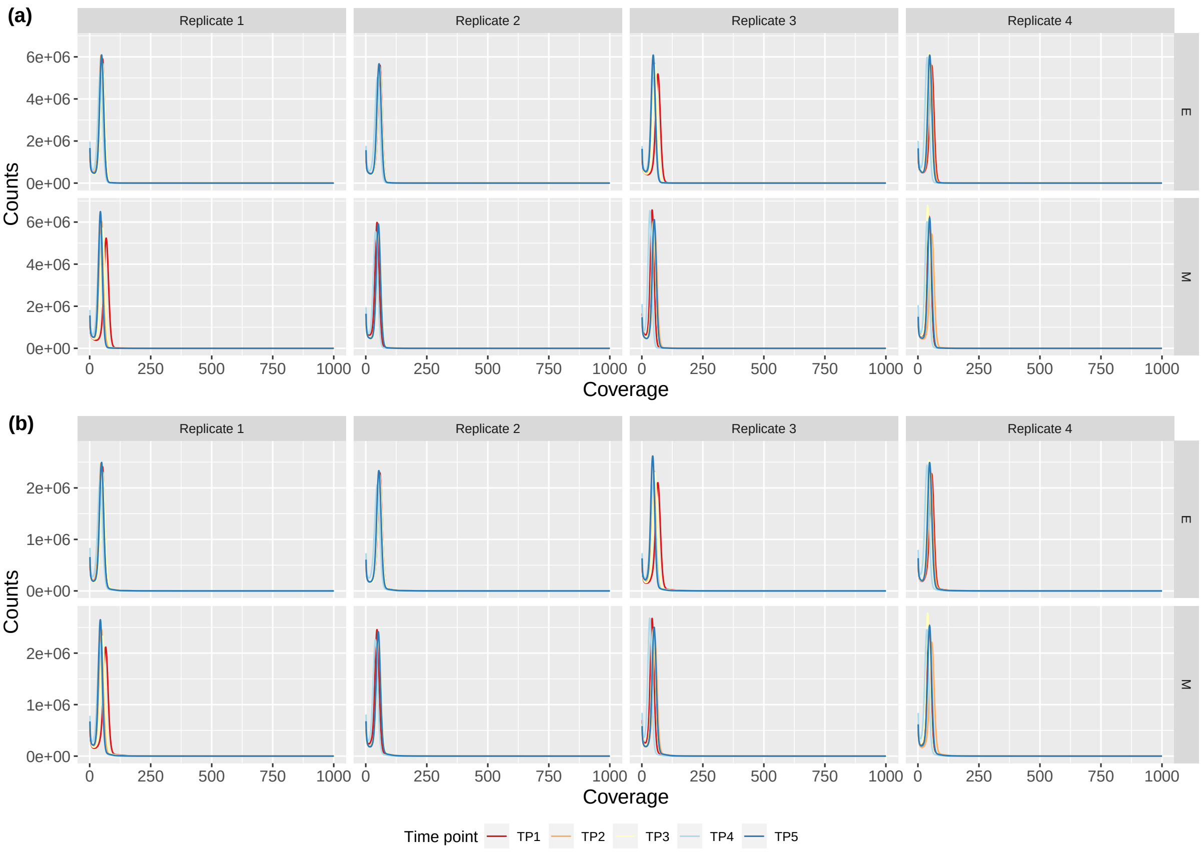

Figure S2: **Read coverage distribution at the genome and X chromosome level assemblies.**

Variant calling and filtering statistics

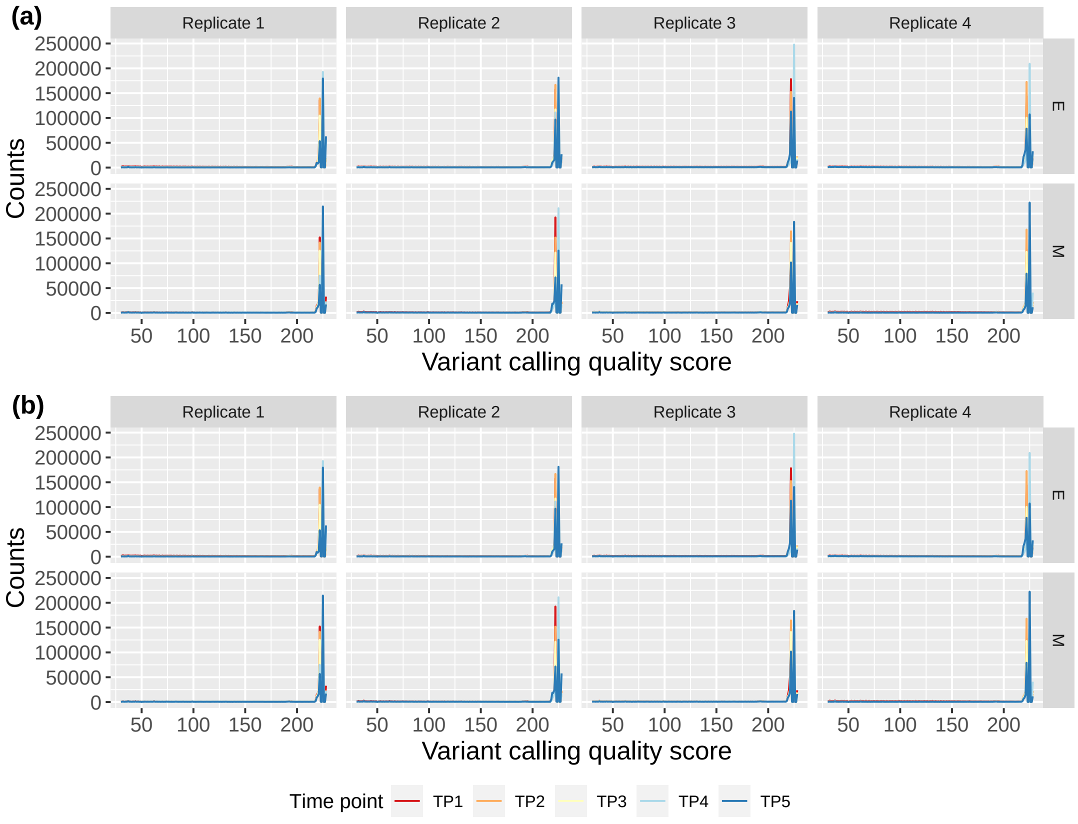

Figure S3: **Variant calling phred quality score per time point for each replicate.**

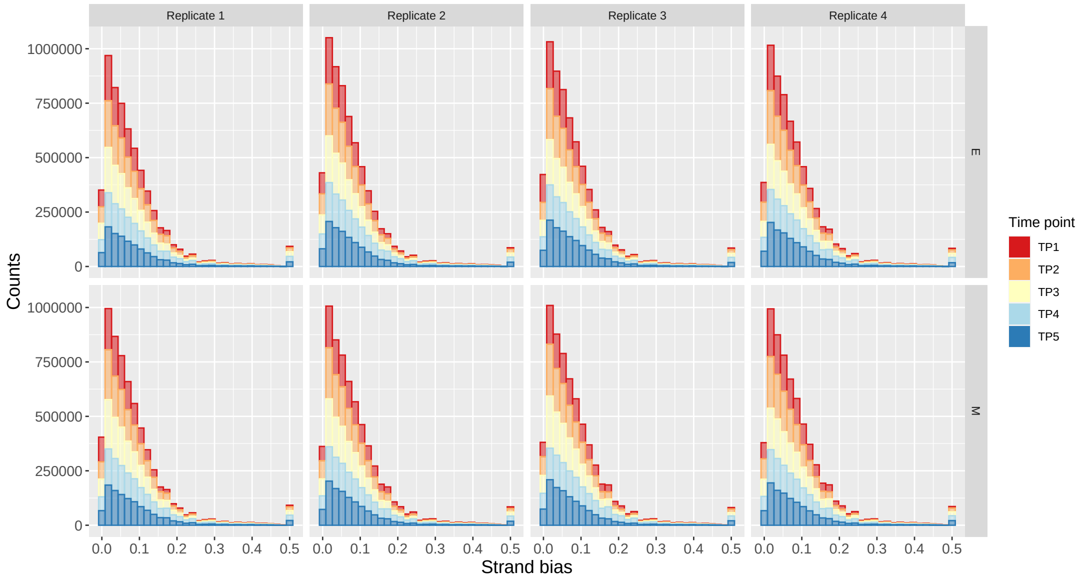

Figure S4: **Strand bias after filtering per time point for each of the four experimental replicates.**

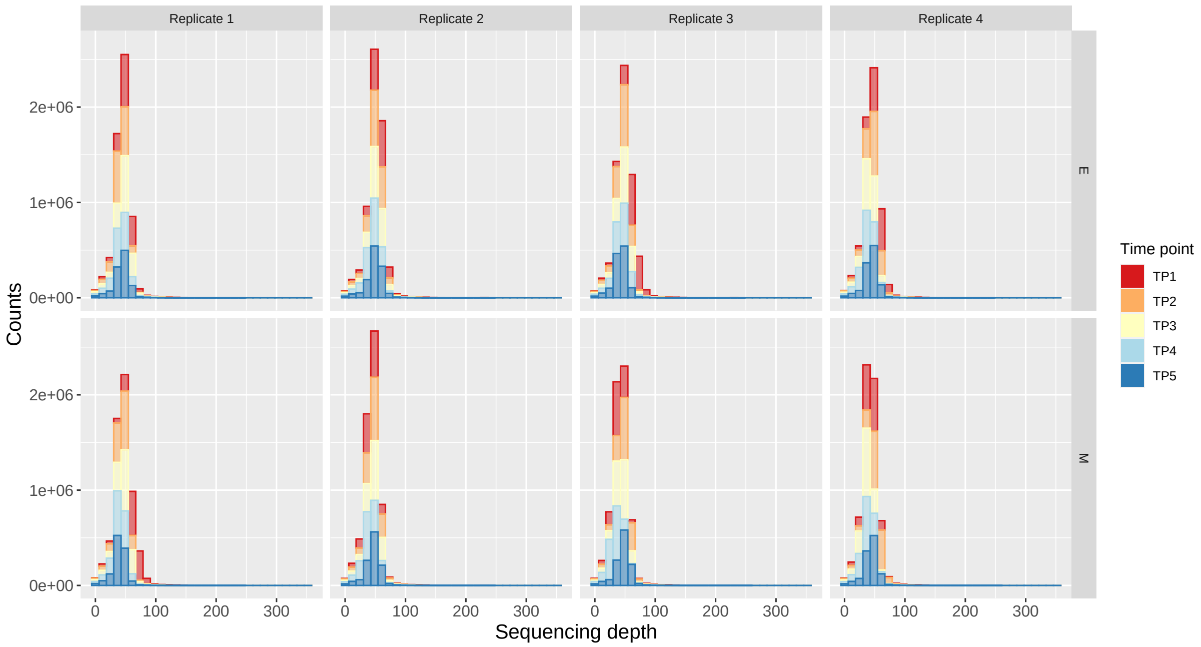

Figure S5: **Sequencing depth distribution per time point for all variants called and retained after filtering.**

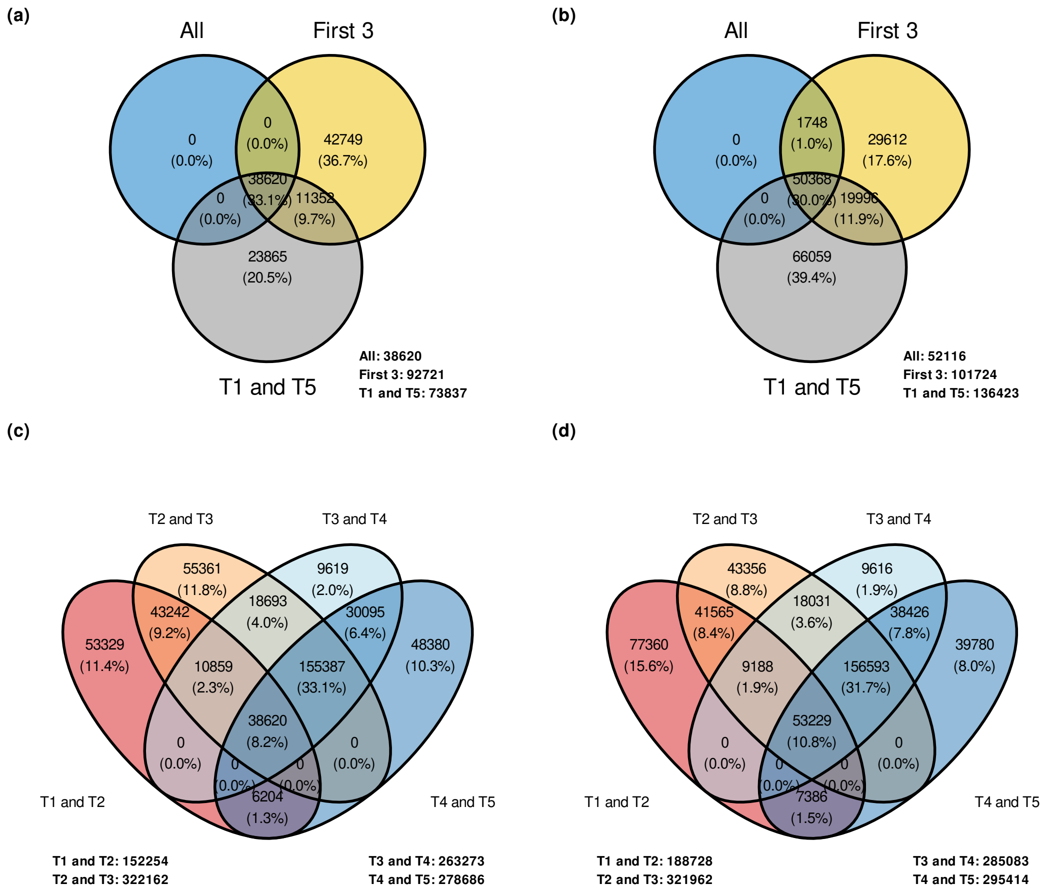

Figure S6: **Venn diagrams that compare the final number of SNPs analysed between different time point interval datasets.**

Treatment	Time point	Unfiltered	Filtered round #1 (vs unfiltered)		Both callers (vs filtered round #1)		Filtered round #2 (vs both callers)		Freebayes (vs unfiltered)		Assembly level
M	TP1	2,787,319	2,477,272	(-310,046.25)	1,466,325	(-1,010,947.75)	1,166,114.75	(-300,209.75)	3,156,979	(+369,660.75)	whole genome
M	TP2	2,491,572	2,303,865	(-187,707)	1,435,954	(-867,911.25)	1,387,945.25	(-48,008.5)	2,305,271	(-186,300.75)	whole genome
M	TP3	2,351,323	2,182,542	(-168,781.5)	1,384,611	(-797,930.75)	1,358,823.50	(-25,787.5)	2,117,929	(-233,394.75)	whole genome
M	TP4	1,967,133	1,847,275	(-119,857.75)	1,240,078	(-607,197.75)	1,204,845.00	(-35,232.5)	1,632,634	(-334,499)	whole genome
M	TP5	1,948,784	1,842,966	(-105,817.75)	1,209,587	(-633,379.25)	1,189,898.50	(-19,688.5)	1,754,641	(-194,142.75)	whole genome
E	TP1	2,843,322	2,547,546	(-295,776)	1,498,669	(-1,048,876.25)	1,214,163.25	(-284,506)	2,910,488	(+67,166.5)	whole genome
E	TP2	2,453,723	2,279,650	(-174,072.75)	1,430,774	(-848,876)	1,386,282.50	(-44,491.5)	2,279,557	(-174,165.75)	whole genome
E	TP3	2,135,627	2,013,440	(-122,186.5)	1,301,792	(-711,648)	1,283,460.00	(-18,332.25)	1,861,553	(-274,074.25)	whole genome
E	TP4	1,910,089	1,802,535	(-107,553.75)	1,210,829	(-591,705.75)	1,176,927.25	(-33,901.75)	1,573,896	(-336,193)	whole genome
E	TP5	2,062,411	1,929,839	(-132,571.5)	1,242,848	(-686,991.5)	1,214,033.25	(-28,814.5)	1,979,512	(-82,898.75)	whole genome
M	TP1	1,047,522	942,455	(-105,067)	565,788	(-376,666.5)	–	–	1,182,418	(-134896)	X
M	TP2	925,122	852,754	(-72,368.5)	528,325	(-324,429)	–	–	900,689	(+24433.25)	X
M	TP3	871,490	804,422	(-67,068)	506,213	(-298209)	–	–	832,029	(+39461)	X
M	TP4	731,140	676,547	(-545,92.75)	450,819	(-225728.5)	–	–	654,219	(+76920.5)	X
M	TP5	739,132	687,412	(-517,19.75)	445,051	(-242361.75)	–	–	726,722	(+12410.25)	X
E	TP1	1,055,999	949,923	(-106,076)	560,418	(-389505)	–	–	1,100,105	(-44105.5)	X
E	TP2	915,083	846,588	(-68,494.5)	530,034	(-316553.75)	–	–	897,880	(+17202.25)	X
E	TP3	777,659	724,817	(-52,841.5)	465,100	(-259717.25)	–	–	733,010	(+44648.25)	X
E	TP4	690,257	642,877	(-47,379.25)	430,364	(-212513.75)	–	–	611,128	(+79128.25)	X
E	TP5	770,680	711,135	(-59,544.75)	452,685	(-258450)	–	–	810,071	(-39391.25)	X

Table S3: **Number of SNPs at different stages of parsing for M and E lines.**

Interval	Treatment	Chromosome 2	Chromosome 3	Chromosome 4	Chromosome X	Total
All time points	M	11128	10537	6593	9807	38065
First three	M	25300	22547	18568	25191	91606
T1T5	M	21084	17144	13186	21396	72810
T1T2	M	43477	33217	30954	42911	150559
T2T3	M	78318	58094	63784	119452	319648
T3T4	M	68139	45920	51847	95260	261166
T4T5	M	71413	45159	57190	102471	276233
All time points	E	12189	14021	11422	13707	51339
First three	E	25358	23347	23278	28438	100421
T1T5	E	32883	26935	27344	47587	134749
T1T2	E	48018	35596	38963	64042	186619
T2T3	E	78890	54384	75011	111092	319377
T3T4	E	70816	46865	68182	96712	282575
T4T5	E	70666	47206	75381	99531	292784

Table S4: **Final number of SNPs per treatment for several time point intervals used for subsequent analyses.**

4 # Supplement to diversity analysis

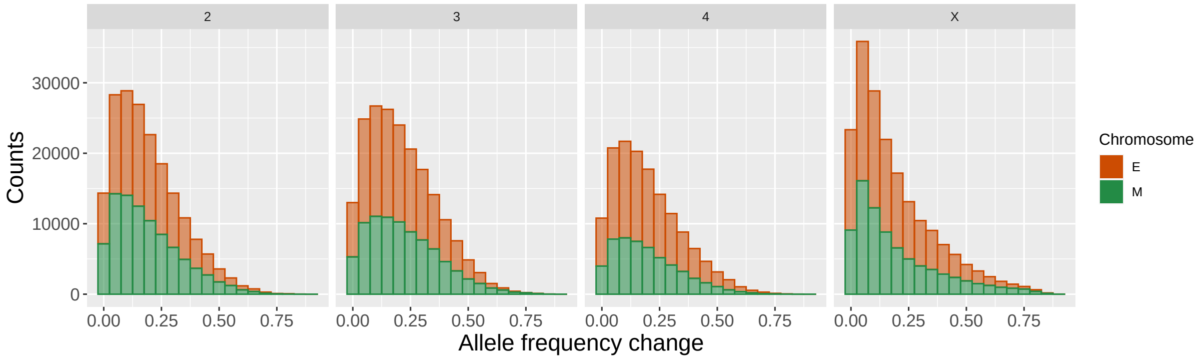

Figure S7: **Allele frequency change histograms in M and E populations for each chromosome.**

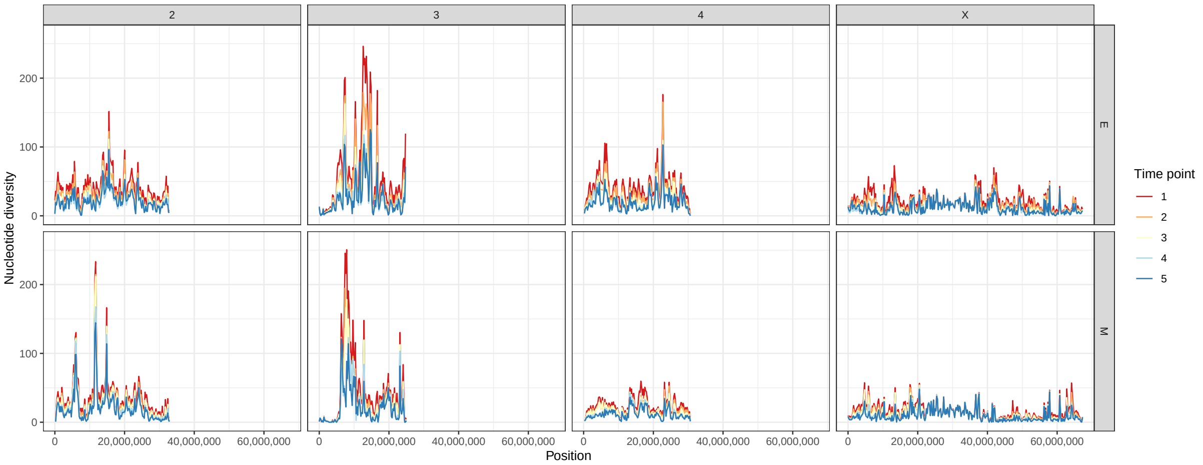

Figure S8: **Average nucleotide diversity, π, along the genome.**

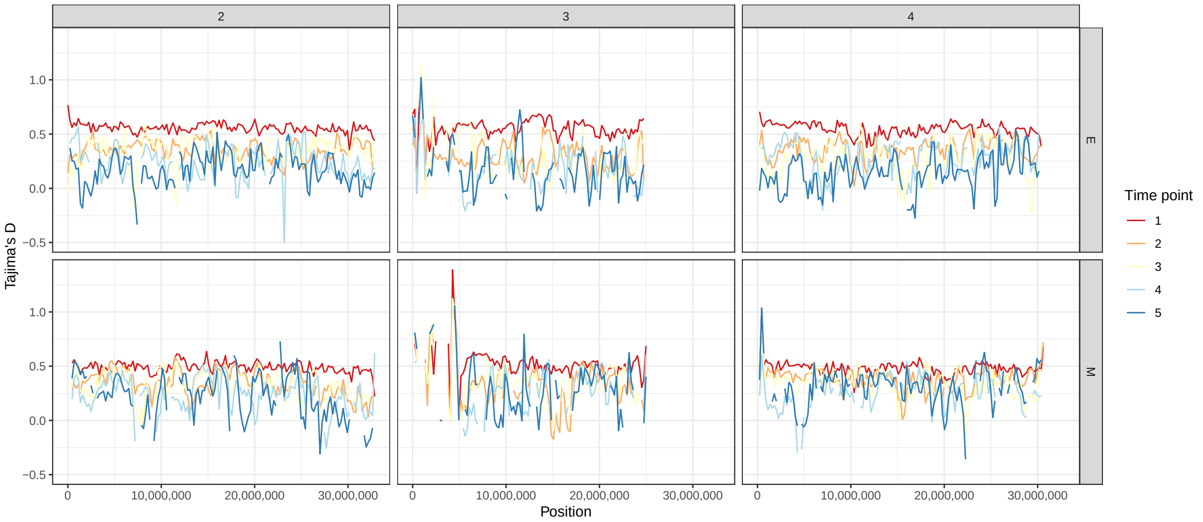

Figure S9: **Tajima's D estimates along chromosomes 2, 3 and 4 for E and M lines.**

Supplement to N_e analysis

Time interval	Median - M	Median - E
Overall	151.0 (n = 100)	159.2 (n = 223)
T1-T2	90.0 (n = 212)	85.7 (n = 308)
T2-T3	68.2 (n = 246)	111.9 (n = 239)
T3-T4	73.8 (n = 131)	102.9 (n = 112)
T4-T5	134.8 (n = 107)	145.8 (n = 116)

Table S5: **Median genome-wide N_e estimates for M and E lines at different time point intervals using intergenic SNPs only.**

Level	Treatment	Replicate	T1-T2	T2-T3	T3-T4	T4-T5	Overall
Genome	E	1	82.06	120.54	133.39	247.12	107.98
Autosomes	E	1	83.49	118.26	110.81	220.65	111.22
Chromosome X	E	1	81.12	122.37	180.87	265.40	80.91
Chromosome 2	E	1	75.48	114.93	149.02	302.58	114.55
Chromosome 3	E	1	86.71	100.60	155.92	192.70	109.20
Chromosome 4	E	1	87.41	152.84	102.94	165.40	95.49
Genome	E	2	92.41	115.91	90.94	123.36	155.18
Autosomes	E	2	101.10	113.50	91.99	123.32	170.87
Chromosome X	E	2	68.52	135.77	75.27	128.90	134.87
Chromosome 2	E	2	112.34	103.41	85.47	129.23	200.52
Chromosome 3	E	2	56.94	117.63	176.10	90.79	169.80
Chromosome 4	E	2	101.60	142.13	95.07	92.03	124.54
Genome	E	3	89.00	101.45	122.78	69.63	144.11
Autosomes	E	3	103.41	131.91	102.53	69.63	168.37
Chromosome X	E	3	69.68	59.41	180.68	59.71	136.07
Chromosome 2	E	3	115.13	162.78	103.89	54.70	190.03
Chromosome 3	E	3	78.63	110.65	102.53	80.85	124.65
Chromosome 4	E	3	86.29	125.42	104.11	82.89	165.32
Genome	E	4	116.57	104.00	117.82	156.75	193.05
Autosomes	E	4	148.46	113.60	113.11	149.36	208.91
Chromosome X	E	4	77.81	92.37	142.05	185.52	147.58
Chromosome 2	E	4	124.70	100.36	109.83	190.79	216.42
Chromosome 3	E	4	256.69	89.73	150.11	152.19	235.55
Chromosome 4	E	4	144.51	147.04	79.27	109.67	197.57

Table S6: **Median N_e estimates for M line replicates at different time point intervals at the genome-wide, autosome-wide and chromosome-wide level.**

Level	Treatment	Replicate	T1-T2	T2-T3	T3-T4	T4-T5	Overall
Genome	M	1	67.41	38.11	119.42	156.27	125.30
Autosomes	M	1	73.40	39.74	120.39	150.19	117.18
Chromosome X	M	1	56.59	37.92	104.62	196.06	141.89
Chromosome 2	M	1	59.78	48.25	104.67	144.12	117.76
Chromosome 3	M	1	71.28	37.75	158.37	221.78	112.81
Chromosome 4	M	1	88.18	37.50	107.57	102.35	116.15
Genome	M	2	83.74	106.98	87.41	122.42	178.90
Autosomes	M	2	94.20	127.23	90.45	134.89	173.27
Chromosome X	M	2	73.35	98.06	78.54	73.47	190.78
Chromosome 2	M	2	85.22	162.71	155.33	98.45	191.41
Chromosome 3	M	2	131.03	97.36	56.28	217.45	156.83
Chromosome 4	M	2	83.74	101.55	94.47	122.42	169.47
Genome	M	3	117.52	80.73	45.53	145.99	163.84
Autosomes	M	3	116.22	82.31	48.02	155.19	171.86
Chromosome X	M	3	127.13	79.69	32.24	98.14	140.24
Chromosome 2	M	3	174.65	128.81	49.52	189.23	199.82
Chromosome 3	M	3	94.58	67.54	30.37	84.02	142.88
Chromosome 4	M	3	113.20	67.63	45.43	148.37	178.32
Genome	M	4	153.76	53.49	33.89	94.77	119.70
Autosomes	M	4	158.42	62.15	34.27	77.58	111.75
Chromosome X	M	4	139.82	45.74	30.13	166.21	144.88
Chromosome 2	M	4	211.34	81.69	34.95	79.12	116.79
Chromosome 3	M	4	104.22	51.73	48.58	87.54	113.94
Chromosome 4	M	4	98.98	55.79	26.02	76.04	106.58

Table S7: **Median N_e estimates for M line replicates at different time point intervals at the genome-wide, autosome-wide and chromosome-wide level.**

Replicate	Treatment	T1-T2	T2-T3	T3-T4	T4-T5	Overall
1	E	0.97	1.03	1.63	1.20	0.73
2	E	0.68	1.20	0.82	1.05	0.79
3	E	0.67	0.45	1.76	0.86	0.81
4	E	0.52	0.81	1.26	1.24	0.71
1	M	0.77	0.95	0.87	1.31	1.21
2	M	0.78	0.77	0.87	0.54	1.10
3	M	1.09	0.97	0.67	0.63	0.82
4	M	0.88	0.74	0.88	2.14	1.30

Table S8: N_{eX}/N_{eA} **ratios for M and E lines at different time point intervals for each replicate population.**

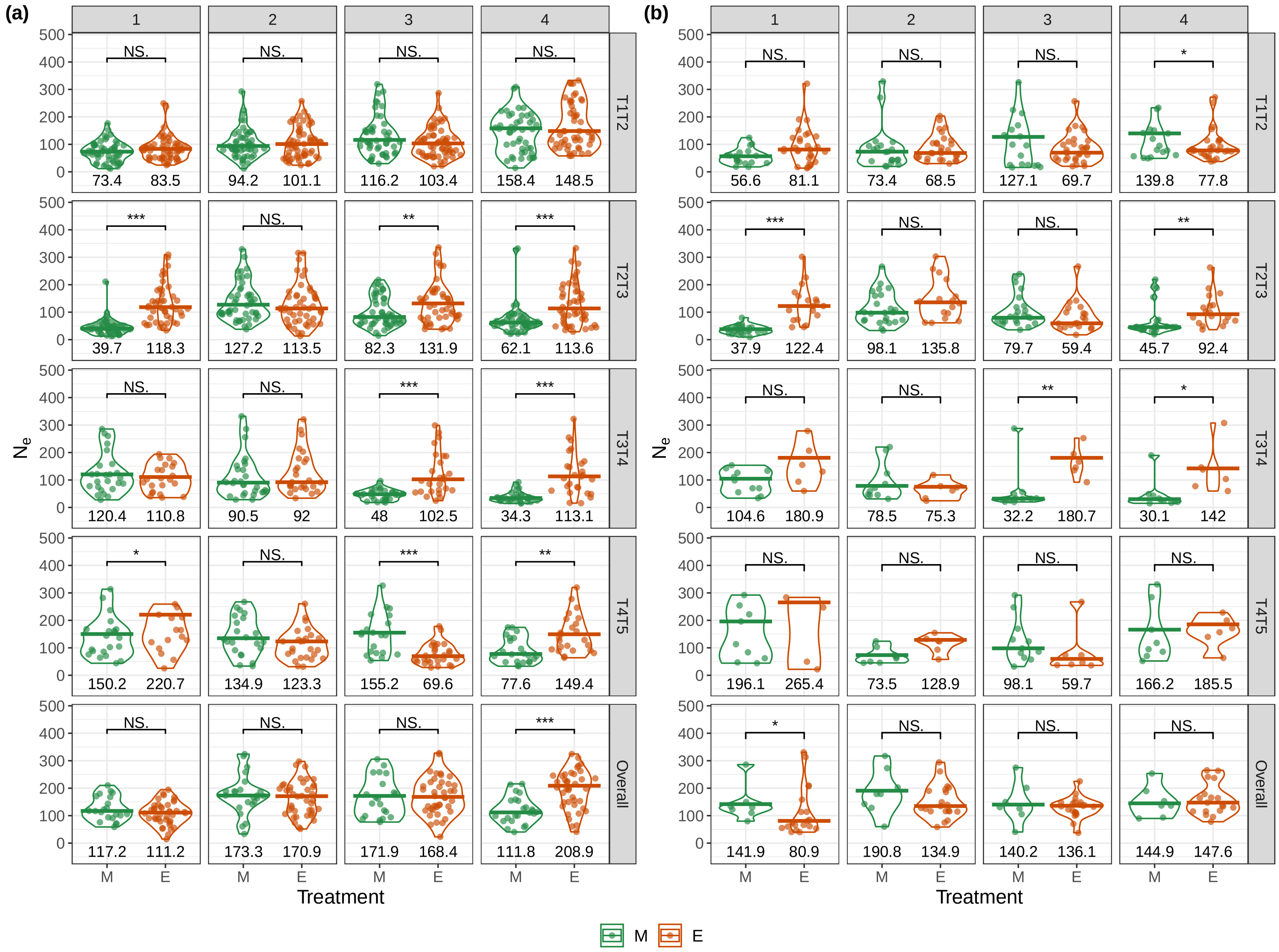

Figure S10: N_e **estimates at the (a) autosome- and (b) X chromosome-level at different time point intervals comparing M and E lines.**

Generalised Additive Model for N_e

We fit a Generalised Additive Model to describe N_e throughout the experimental evolution. We assigned 'replicate' as a random effect and 'time-point' was defined as a predictor variable modelled with spline regression (with 3 knots) for each treatment. We filtered the N_e data to remove nonsensical values, i.e., negative estimates and estimates that are one order of magnitude above the median. Filtered out estimates are computed for windows with too few SNPs to generate reliable estimates. Below we provide a summary of the model results as well as the fitted curves for each treatment.

```
####################### Model summary output #######################
Family: gaussian
Link function: identity

Formula:
estimates ~ s(tp_val, by = treatment, bs = "tp", k = 3) + s(replicate,
    bs = "re")

Parametric coefficients:
            Estimate Std. Error t value Pr(>|t|)
(Intercept)  132.523      4.605   28.77   <2e-16 ***
---
Signif. codes:  0 '***' 0.001 '**' 0.01 '*' 0.05 '.' 0.1 ' ' 1

Approximate significance of smooth terms:
                        edf Ref.df      F p-value
s(tp_val):treatmentE 1.014  1.029 10.571 0.00113 **
s(tp_val):treatmentM 1.964  1.999 16.826 < 2e-16 ***
s(replicate)         1.742  3.000  1.389 0.06687 .
---
Signif. codes:  0 '***' 0.001 '**' 0.01 '*' 0.05 '.' 0.1 ' ' 1

R-sq.(adj) =  0.0261   Deviance explained = 2.87%
-REML =  11057  Scale est. = 15705     n = 1770
####################################################################
```

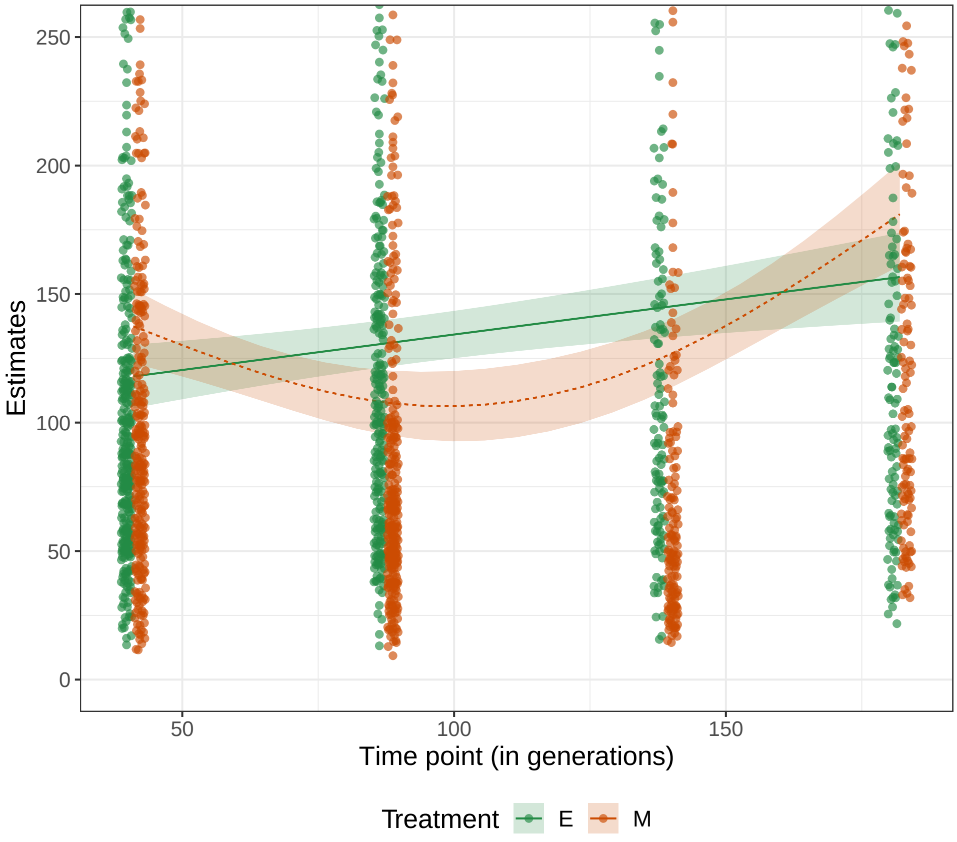

Figure S11: **Fitted GAM curves of N_e for E and M treatments.**

Supplement to genome scan

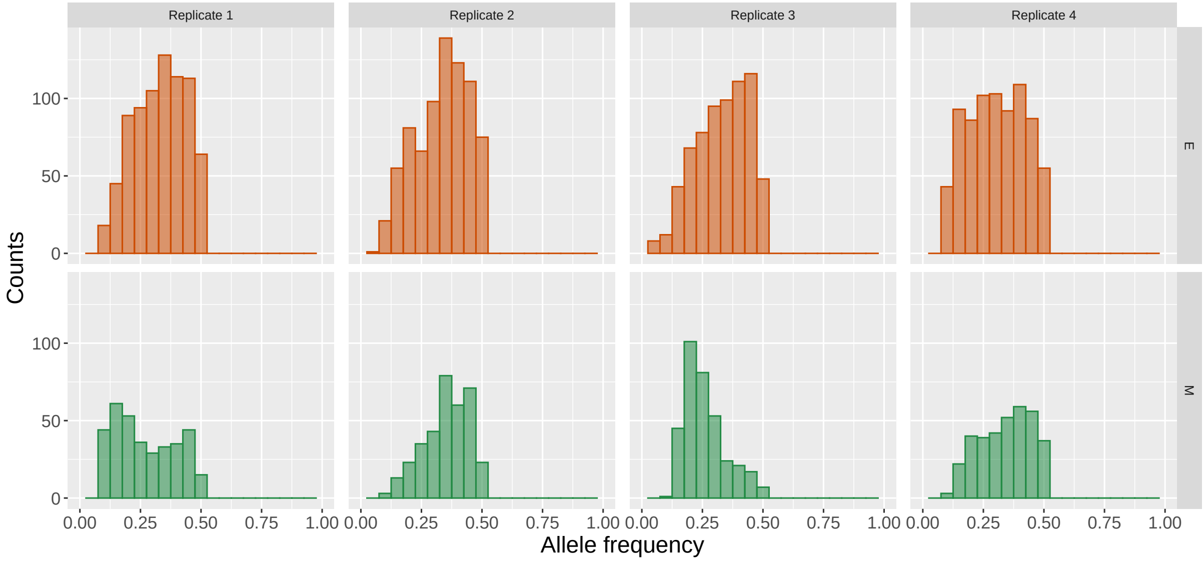

Figure S12: **Starting allele frequency of target loci found in genome scan.**

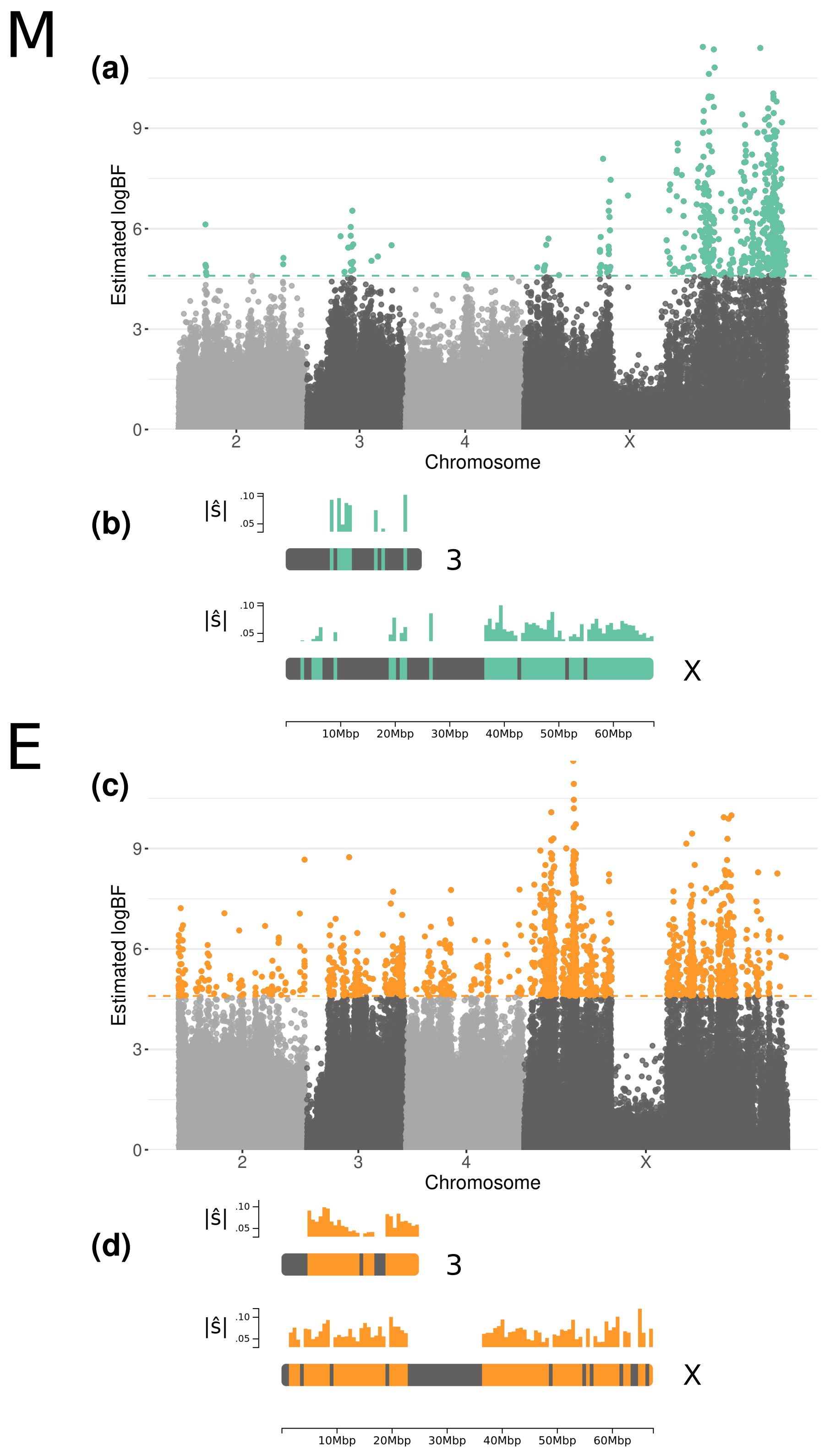

Figure S13: **Genome scan for signatures of adaptation throughout the genome for M (top) and E (bottom) lines for the first half of the experiment.**

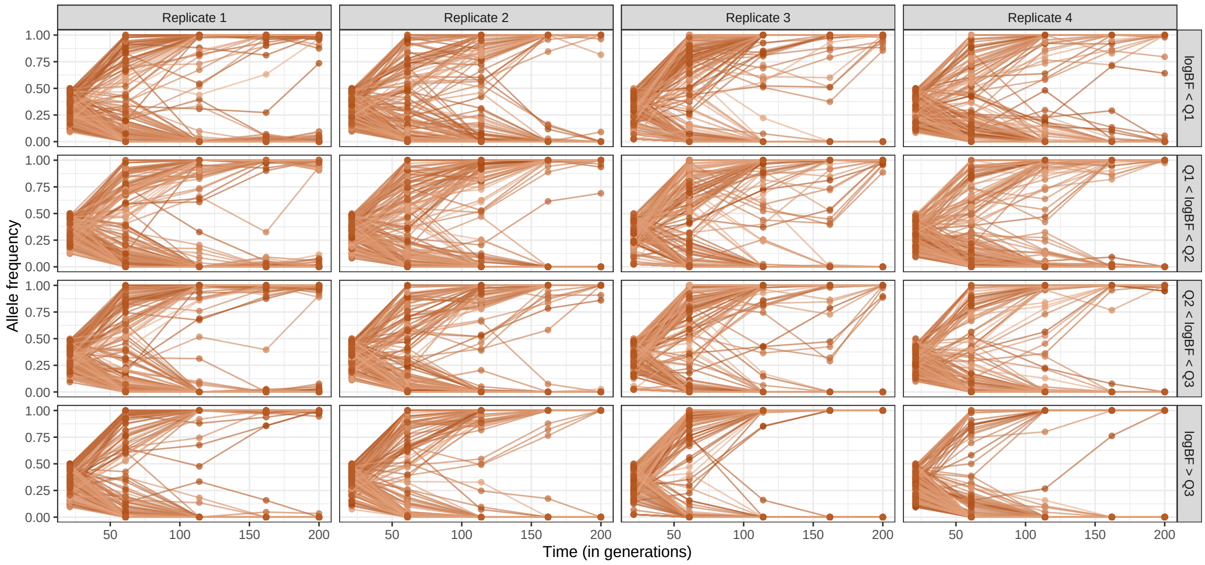

Figure S14: **Five time point allele frequency trajectories of selected loci in E populations.**

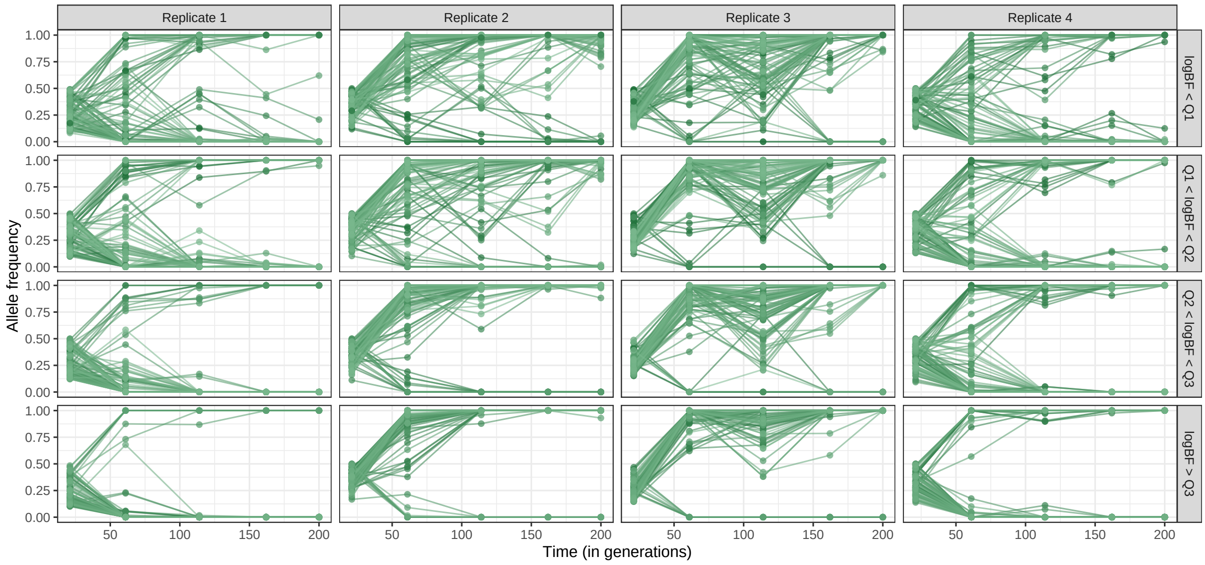

Figure S15: **Five time point allele frequency trajectories of selected loci in M populations.**

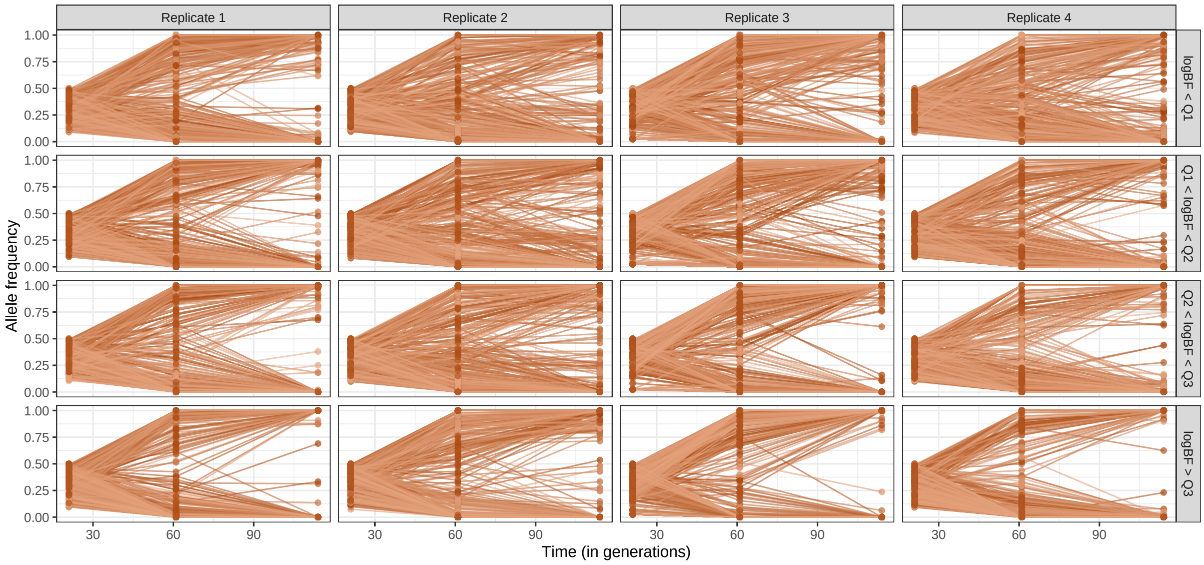

Figure S16: **Three time point allele frequency trajectories of selected loci in E populations.**

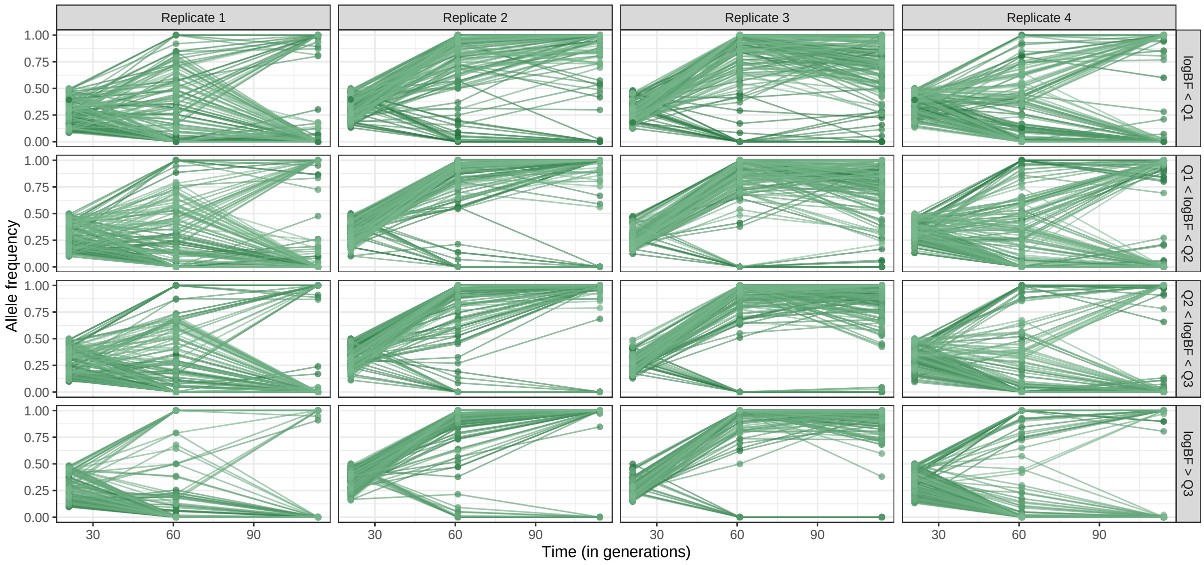

Figure S17: **Three time point allele frequency trajectories of selected loci in M populations.**

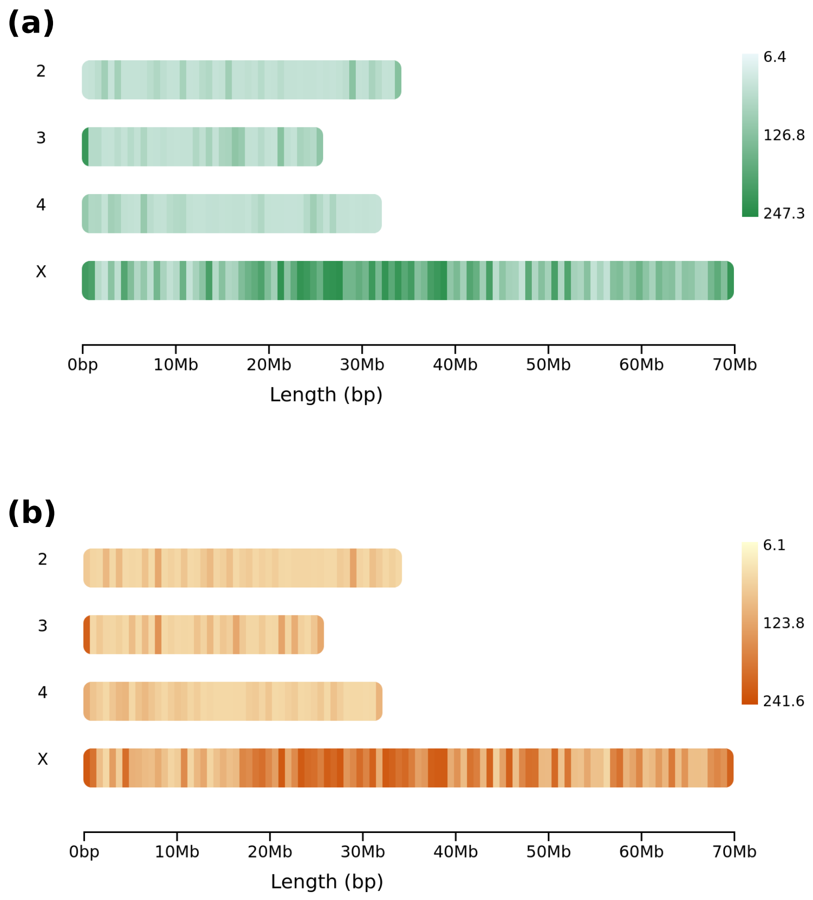

Figure S18: **Maximum coverage chromosome plots for (a) M and (b) E lines.**

Treatment	Chromosome	Location	NCBI ID	FB ID	*D. mel* ortholog	Gene name	#top SNPs	Distance
E	2	Gene	6896726	FBgn0262041	hdc	headcase protein	1	NA
E	3	Gene	4805669	FBgn0073138	SmydA-5	SET domain-containing protein SmydA-8	1	NA
E	3	Gene	4805666	FBgn0080959	Cyp4aa1	cytochrome P450 4aa1	1	NA
E	3	Gene	6899100	FBgn0246519	Pgant9	polypeptide N-acetylgalactosaminyltransferase 9	1	NA
E	3	Gene	6899112	FBgn0246524	Verprolin 1	WAS/WASL-interacting protein family member 3	1	NA
E	3	Gene	6899088	FBgn0263807	Strn-Mlck	myosin light chain kinase	2	NA
E	X	Gene	4815279	FBgn0075585	fzr	fizzy-related protein homolog	3	NA
E	X	Gene	4814581	FBgn0078871	forked	espin	3	NA
E	X	Gene	4815373	FBgn0080302	CG7378	dual specificity protein phosphatase 3	1	NA
E	X	Gene	4814961	FBgn0081931	CG9657	sodium-coupled monocarboxylate transporter 1	1	NA
E	X	Gene	4814480	FBgn0243547	Atg5	autophagy protein 5	4	NA
E	X	Gene	6901434	FBgn0247979	dpr8	zwei Ig domain protein zig-8	5	NA
M	X	Gene	6902114	FBgn0244416	CG32532	homeobox protein Hmx	1	NA
E	3	Intergenic region	4805672	FBgn0073140	CCHa2-R	neuropeptide CCHamide-2 receptor	1	-421
E	3	Intergenic region	6899092	FBgn0245480	resilin	pro-resilin	2	-579
E	4	Intergenic region	4816335	FBgn0077513	CG3528	cilia- and flagella-associated protein 299	1	-9236
E	X	Intergenic region	4815048	FBgn0074111	unc-119	protein unc-119 homolog	1	2760
E	X	Intergenic region	4814454	FBgn0080287	Flacc	fl(2)d-associated complex component	1	1833
E	X	Intergenic region	4814479	FBgn0081927	brinker	J domain-containing protein DDB_G0295729	1	-3996
E	X	Intergenic region	6901756	FBgn0249747	CG15034	uncharacterised protein	1	-516
E	X	Intergenic region	6901755	FBgn0249791	-	antigen 5 like allergen Cul n 1	1	1613

Table S9: **Common genes amongst top scoring variants in this study and Wiberg et al. (2021).**

Chromosome	Treatment	# top SNPs	Gene NCBI ID	Gene FB ID	*D. mel* ortholog	Gene name
X	E	7	4813031	FBgn0076699	Lmx1a	LIM homeobox transcription factor 1-beta
X	E	9	4813494	FBgn0076929	SpoCk	calcium-transporting ATPase type 2C member 1
X	E	11	4813557	FBgn0076932	neuromusculin	hemicentin-1
X	E	6	4814416	FBgn0077610	Fas2	fasciclin-2
X	E	5	4814942	FBgn0079214	Nep1	neprilysin-1
3	E	5	6899052	FBgn0263814	jing	zinc finger protein jing
3	E	5	6899426	FBgn0250039	luna	Krueppel-like factor luna
X	E	23	6901139	FBgn0243670	CG43867	uncharacterised protein
X	E	5	6901434	FBgn0247979	dpr8	zwei Ig domain protein zig-8
X	E	7	6901448	FBgn0247929	Sh	potassium voltage-gated channel protein Shaker
X	E	7	6901459	FBgn0247897	CG5921	uncharacterised protein
X	E	5	6901716	FBgn0245264	-	voltage-dependent T-type calcium channel subunit alpha-1G
X	E	9	6901746	FBgn0245096	SK	small conductance calcium-activated potassium channel protein

Table S10: **Genes with the most significant variants.**

List of Supplementary Figures

S1 **Mapping quality score distribution at the genome (a) and X chromosome (b) level assemblies.** Rows correspond to the two treatments, E (top) and M (bottom), and columns to the four experimental replicates. Each time point is coloured differently as per legend at the bottom. 2

S2 **Read coverage distribution at the genome and X chromosome level assemblies.** Each column corresponds to an different replicate population and each row to either E (top) or M (bottom) lines. The top panel - (a) - shows genome level data and the bottom panel - (b) - X chromosome data. Different time points are coloured according to legend at the bottom of the figure. 3

S3 **Variant calling phred quality score per time point for each replicate.** (a) shows quality score distributions at the genome level and (b) at the X chromosome level assemblies. Each row corresponds to a different treatment - E on top row and M on bottom - and each column to an individual replicate. Time points are visible by differently coloured lines as per legend at the bottom of the graph. 4

S4 **Strand bias after filtering per time point for each of the four experimental replicates.** E populations are on the top tow, and M on the bottom. Each column corresponds to an individual replicate. Time point distributions are coloured differently as per side legend. 4

S5 **Sequencing depth distribution per time point for all variants called and retained after filtering.** Replicates can be found in columns, and treatments in rows (E: top, M: bottom). Distributions for each time point were coloured differently as per side legend. 5

S6 **Venn diagrams that compare the final number of SNPs analysed between different time point interval datasets.** Panels (a) and (b) show number of SNPs found in a five time point time series ('All'), a three time point time series ('First 3') or the first and last time points (T1 and T5) for M and E, respectively. Bottom panels (c) – M lines – and (d) – E lines – compare two time point intervals: T1 and T2, T2 and T3, T3 and T4, and T4 and T5. . . 5

S7 **Allele frequency change histograms in M (in green) and E (in orange) populations for each chromosome (columns).** These are calculated as the difference in allele frequency between first and last time point for each individual SNP. 7

S8 **Average nucleotide diversity, π, along the genome.** Columns correspond to chromosomes and rows to the two different treatments (top: E; bottom: M). Lines are coloured as to show variation across time. Averages were calculated across replicates for each 250k SNP window separately. 8

S9 **Tajima's D estimates along chromosomes 2, 3 and 4 for E and M lines.** Rows correspond to the two different treatments and columns to chromosomes. Estimates were calculated in 250k SNP windows with Grenedalf (Czech and Exposito-Alonso, 2021). Lines are coloured per time point according to the side legend. 8

S10 **N_e estimates at the (a) autosome- and (b) X chromosome-level at different time point intervals comparing M and E lines.** Violin plots and data points are included for each time point interval and replicate per treatment. Outliers were removed. Medians are shown as bars and median estimates can be found at the bottom of each violin plot. These were calculated using all 2k SNP window estimates from the four experimental replicates. 'Overall' corresponds to N_e estimates based on allele frequency changes between the first and last time point. Adjacent time point intervals were compared using a Mann-Whitney U test. Significance level is indicated with *** for p-value $<$ 0.001, ** for p-value $<$ 0.01, * for p-value $<$ 0.05, and N.S. is non-significant. 12

S11 **Fitted GAM curves of N_e for E and M treatments.** Orange: E lines. Green: M lines. Data points were included to demonstrate the variability in N_e estimates. 14

S12 **Starting allele frequency of target loci found in genome scan.** Histograms for individual replicates are found in each column (1 to 4) and rows represent E (top) and M (bottom) treatment lines. 15

S13 **Genome scan for signatures of adaptation throughout the genome for M (top) and E (bottom) lines for the first half of the experiment.** (a) and (c) are manhattan plots of Bait-ER (Barata et al., 2020) logBF for each allele considering frequency trajectories that span over the first three time points. Statistically significant SNPs are coloured in green (M, top) or orange (E, bottom). Dashed lines correspond to a threshold of $log(99) \approx 4.6$. (b) and (d) are diagrams of chromosomes 3 (top) and X (bottom) that illustrate which regions of each chromosome harboured the most number of significant hits. Average estimated selection coefficients ($|\widehat{s}|$) for each interval can be found above each diagram as a bar plot. Data excludes chromosome 5. 16

S14 **Five time point allele frequency trajectories of selected loci in E populations.** Individual trajectories until generation 200 where each column represents a different replicate population, and each rows a quantile of the logBF values computed in our genome scan. 17

S15 **Five time point allele frequency trajectories of selected loci in M populations.** Individual trajectories until generation 200 where each column represents a different replicate population, and each rows a quantile of the logBF values computed in our genome scan. 17

S16 **Three time point allele frequency trajectories of selected loci in E populations.** Individual trajectories until generation 200 where each column represents a different replicate population, and each rows a quantile of the logBF values computed in our genome scan. 18

S17 **Three time point allele frequency trajectories of selected loci in M populations.** Individual trajectories until generation 200 where each column represents a different replicate population, and each rows a quantile of the logBF values computed in our genome scan. . . . 18

S18 **Maximum coverage chromosome plots for (a) M and (b) E lines.** Each diagram represents the maximum coverage of any given interval in each of the four chromosomes analysed. M lines are coloured in green and E in orange. Highest coverage regions can indicate repetitive elements and are common in telomeres and centromeres. Data exclude chromosome 5. 19

List of Supplementary Tables

S1 **Description of which generations were sampled at each time point.** Both M and E lines were sampled at the same generation for each corresponding replicate population. TP: time point. 1

S2 **Mapping statistics for both mappers.** This includes average number of mapped reads as well as the average percentage across samples for M and E lines. Data on whole genome and X chromosome level assemblies can be found on this table. 1

S3 **Number of SNPs at different stages of parsing for M and E lines.** 'Unfiltered' are the average total number of SNPs at each time point called by bcftools before any parsing. Subsequent filtering for variants called with a quality score of at least 30 resulted in the 'Filtered round #1' column, where numbers in brackets are the number of SNPs lost in this parsing step. Only SNPs that were called by both bcftools and Freebayes were retained after the first filtering step - 'Both callers'. Variants lost here are in brackets. 'Filtered round #2' included keeping biallelic sites only, as well as retaining solely those variants that were called both in the bwa mem and the novoalign alignment. In first time point samples, only polymorphic sites with a MAF $\geq$ 0.025 were kept. SNPs called by Freebayes are included for comparison ('Freebayes'). Figures in brackets here are the difference to 'Unfiltered' polymorphisms. 6

S4 **Final number of SNPs per treatment for several time point intervals used for subsequent analyses.** 'All time points' and 'First three' correspond to the two time series we have analysed which have information on either 5 or 3 time points, respectively. T1T5, T1T2, T2T3, T3T4 and T4T5 are every interval combination of any two time points. Total SNP numbers for chromosomes 2, 3, 4 and X can be found in columns 3 to 6. 7

S5 **Median genome-wide N_e estimates for M and E lines at different time point intervals using intergenic SNPs only.** Medians were calculated using 1k intergenic SNP window estimates from all of the four experimental replicates. 'Overall' corresponds to N_e estimates based on allele frequency changes between the first and last time point. The total number of windows considered in each replicate is found in brackets. 8

S6 **Median N_e estimates for E line replicates at different time point intervals at the genome-wide, autosome-wide and chromosome-wide level.** Medians were calculated using 2k SNP window estimates from all of the four experimental replicates. 'Overall' corresponds to Ne estimates based on allele frequency changes between the first and last time point. Coloured cells indicate minimum (in purple) and maximum (in yellow) estimates throughout the time series for each replicate. 9

S7 **Median N_e estimates for M line replicates at different time point intervals at the genome-wide, autosome-wide and chromosome-wide level.** Medians were calculated using 2k SNP window estimates from all of the four experimental replicates. ‘Overall‘ corresponds to Ne estimates based on allele frequency changes between the first and last time point. Coloured cells indicate minimum (in purple) and maximum (in yellow) estimates throughout the time series for each replicate. 10

S8 **N_{eX}/N_{eA} ratios for M and E lines at different time point intervals for each replicate population.** 'Overall' corresponds to N_e estimates based on allele frequency changes between the first and last time point. 11

S9 **Common genes amongst top scoring variants in this study and Wiberg et al. (2021).** This includes data on genes where significant variants are located in or genes near top SNPs. Each gene is described by an NCBI ID, a FlyBase ID, a *D. melanogaster* ortholog, the gene's name, how many top SNPs mapped on to the gene or the distance between any intergenic SNPs and the nearest gene. 20

S10 **Genes with the most significant variants.** Each gene is described by the number of top SNPs located in said gene, an NCBI ID, a FlyBase ID, a *D. melanogaster* ortholog and the gene's name. 21
